# Antibiotic prescribing appropriateness among critically ill patients at a single tertiary care center in the United Arab Emirates: a retrospective cohort study

**DOI:** 10.3389/frhs.2026.1900610

**Published:** 2026-09-30

**Authors:** Inas Kassis, Mosab Al Balas, Sheilla Hilal Hamed Rashid, Shabaz Mohiuddin Gulam, Jisha Myalil Lucca, Kimberly Caye McKeirnan, Vineetha Menon

**Affiliations:** 1 Department of Pharmacy Practice, College of Pharmacy, Gulf Medical University, Ajman, United Arab Emirates; 2 Clinical Pharmacy Department, Sheikh Tahnoon Bin Mohammad Medical City, Abu Dhabi, United Arab Emirates; 3 Clinical Pharmacy Department, Thumbay University Hospital, Ajman, United Arab Emirates; 4 Department of Pharmacotherapy, College of Pharmacy and Pharmaceutical Sciences, Washington State University, Pullman, WA, United States

**Keywords:** antibacterial agents, antimicrobial stewardship, guideline adherence, intensive care units, treatment outcome

## Abstract

**Objective:**

Despite expanding antimicrobial stewardship initiatives, evidence evaluating the appropriateness of antibiotic prescribing and its clinical implications in intensive care settings in the United Arab Emirates remains limited. This study aimed to assess the appropriateness of antibiotic prescribing among intensive care unit patients, identify factors associated with appropriate antibiotic therapy, and examine its association with clinical outcomes.

**Method:**

A retrospective cohort study was conducted in the intensive care unit of a tertiary care hospital in the Emirate of Abu Dhabi. Adult patients aged 18 years or older who were admitted between January 2023 and December 2024, had a length of stay exceeding 24 h but less than 30 days, received at least one systemic antibiotic, and had complete medical records were included. Data were extracted from electronic medical records and a regional health information exchange platform. Antibiotic appropriateness was evaluated against institutional or Infectious Diseases Society of America guidelines.

**Results:**

Overall, 36% of patients received inappropriate antibiotic therapy. The most common reason for inappropriateness was the absence of a clear indication, particularly for postoperative prophylaxis (44.4%). At the individual agent level, cefepime (48.2%), metronidazole (36.2%), and cefazolin (33.9%) exhibited the highest rates of inappropriate use. Multivariable logistic regression identified positive microbiological culture results (AOR = 3.34, *p* < 0.001) and sepsis (AOR = 2.12, *p* = 0.002) as factors independently associated with higher odds of appropriate antibiotic use. Conversely, the presence of a central venous catheter (AOR = 0.30, *p* = 0.003) was associated with lower odds of appropriate antibiotic use. Appropriate antibiotic therapy was associated with higher odds of organ dysfunction (AOR = 1.69, *p* = 0.032), nosocomial infection (AOR = 2.28, *p* = 0.005), and persistent infection despite therapy (AOR = 2.13, *p* = 0.005).

**Conclusion:**

Approximately one-third of patients received inappropriate antibiotic therapy, representing a substantial antimicrobial stewardship concern despite the observed strengths in culture-guided therapy and infection-focused management.

## 1 Introduction

Antimicrobial resistance (AMR) has emerged as a major global public health threat, with an increasing number of deaths directly or indirectly attributable to resistant infections. According to the World Health Organization (WHO), approximately 1.27 million deaths in 2019 were directly attributable to bacterial AMR, while an estimated 4.95 million deaths were associated with bacterial AMR worldwide (1, 2). Patterns of antimicrobial use within healthcare settings play a central role in the development and spread of resistance, with inappropriate antibiotic prescribing being a key contributor to increased morbidity and mortality and the emergence of resistant pathogens globally (3–5).

Patients admitted to intensive care units (ICUs) are estimated to be five to ten times more susceptible to infections than those in general wards or nonhospital settings, largely because of the complexity and critical nature of their underlying conditions (6). Antibiotic prescribing in ICUs is influenced by multiple factors, including physicians’ prescribing behaviors (3–7), prolonged durations of therapy (8–10), the absence of definitive infection markers (11), and patient-related characteristics such as comorbidities, prior hospital admissions, invasive surgical procedures, microbiological culture results, and previous antibiotic exposure (12–14). These challenges often require intensivists to make clinical assessments and treatment decisions within a narrow time frame, increasing the risk of inappropriate antibiotic initiation, continuation, or escalation (15–17).

Previous studies assessing antibiotic use in ICUs have reported substantial variability in the prevalence of inappropriate prescribing. Studies from low- and middle-income countries have documented higher rates of irrational antibiotic use in ICUs, ranging from 43% to 77.2% (8, 18, 19), whereas relatively lower rates, between 29.8% and 44%, have been reported in upper-middle- and high-income countries (10, 20). Importantly, the implementation of antimicrobial stewardship programs (ASPs) within healthcare facilities has consistently been associated with improved physician adherence to evidence-based infection management guidelines and more judicious antibiotic prescribing (10, 20).

In the United Arab Emirates (UAE), several national initiatives have been undertaken to optimize antibiotic use and mitigate AMR, including the adoption of ASP frameworks across healthcare institutions (21), the enforcement of regulatory restrictions on the dispensing of antibiotics in community pharmacies (22), and the establishment of the UAE National AMR Surveillance System to monitor resistance trends nationwide (23–25). Despite these efforts, existing studies have largely focused on hospital-wide antibiotic utilization, with ICU data often aggregated with those of other patient populations and without a detailed evaluation of prescribing appropriateness, its associated factors, or clinical outcomes (5, 26–28). Consequently, the present study was conducted to assess the appropriateness of antibiotic prescribing, identify factors associated with appropriate antibiotic therapy, and examine the association between antibiotic appropriateness and clinical outcomes among ICU patients at a tertiary hospital in Abu Dhabi.

## 2 Materials and methods

### 2.1 Study design and setting

This retrospective cohort study was conducted at a large tertiary care hospital operating under the Abu Dhabi Health Services Company and PureHealth management systems in the Emirate of Abu Dhabi, UAE. The ICU comprises 20 beds, including two isolation rooms and six rooms equipped for renal replacement therapy (RRT), and is supported by integrated electronic medical record (EMR) and laboratory information systems.

### 2.2 Antimicrobial stewardship

The hospital has an established multidisciplinary ASP committee responsible for reviewing international guidelines and adapting them into institution-specific protocols for antibiotic use. Clinical pharmacists play an integral role in developing, periodically reviewing, and updating these protocols and participate in key stewardship activities, including formulary restriction, prospective audit and feedback, prescribing surveillance, interdisciplinary interventions, and daily ward rounds. These activities support evidence-based antimicrobial use and optimized prescribing practices among critically ill patients.

### 2.3 Study population

Clinical records of patients admitted to the ICU between January 2023 and December 2024 were retrieved and screened according to predefined inclusion and exclusion criteria. Patients aged 18 years or older who were admitted to the ICU for more than 24 h and received at least one systemic antibiotic during their ICU stay were included. Patients with ICU stays of <24 h were excluded because antibiotic exposure, treatment response, and microbiological evaluation could not be reliably assessed within this limited timeframe. Patients with ICU stays of >30 days were excluded to avoid the disproportionate influence of prolonged critical illness, which is characterized by recurrent or multidrug-resistant infections and highly individualized antibiotic management.

Missing data were handled using a complete-case approach. Patients with incomplete records or missing critical information required for analysis were excluded during the data collection phase and were not included in the final analytical dataset. Detailed variable-level quantification of missingness was unavailable because of the retrospective nature of the dataset.

### 2.4 Study tool and variables

A standardized data collection form was developed by the research team to ensure consistent and comprehensive extraction of information from patient medical records. The form was adapted from previously published antimicrobial stewardship (AMS) and ICU antibiotic appropriateness studies (8–10, 18–20, 29–31). It was subsequently reviewed by four investigators with expertise in infectious diseases, AMS, and clinical research to assess its content, clarity, and completeness. Revisions were made based on their feedback before the commencement of data extraction. The form captured variables including patient sociodemographic and clinical characteristics, laboratory and microbiological indices, antibiotic therapy details, AMS indicators, and clinical outcomes.

Sociodemographic variables included age, gender, body mass index (BMI), and ethnicity. Clinical variables encompassed the reason for ICU admission, comorbidities, and lifestyle factors (including smoking status, alcohol use, and drug abuse), presence of central venous catheter, mechanical ventilation, intubation, vasopressor use, systemic corticosteroid therapy, nutritional support, sedative use, and RRT. Clinical history within the 90 days preceding ICU admission was also recorded, including prior hospitalization, prior invasive procedures or surgeries, prior microbiological culture results, and prior systemic antimicrobial exposure. Laboratory and microbiological variables included body temperature, hematologic parameters, inflammatory markers, renal function indices, blood glucose, mean arterial pressure, culture and susceptibility results, infection type, and isolated microorganisms. Antibiotic therapy-related variables comprised prescribed agents, indication, route of administration, dosage, dosing frequency, duration of therapy, therapeutic drug monitoring (where applicable), and modifications such as escalation, de-escalation, optimization, or discontinuation. AMS-related variables included assessments of the appropriateness of indication, empiric therapy selection, antibiotic choice, dosage and dose adjustment, route and frequency of administration, duration of therapy, consideration of microbiological susceptibility results, and therapeutic drug monitoring (where indicated). Clinical outcomes included development of organ dysfunction, nosocomial infection, persistent infection despite therapy, adverse drug events, length of ICU stay, discharge disposition, and mortality.

### 2.5 Data collection

Following the development of the standardized data collection tool, all ICU admission and antibiotic order records for the study period were retrieved from the hospital's EMR system. To ensure that each patient encounter was represented only once in the sampling frame, repeated antibiotic orders within the same admission were consolidated. Readmissions occurring within 24 hours of discharge were considered continuations of the initial admission and merged into a single clinical episode because this short interval was considered to reflect continuation of the same unresolved clinical condition rather than a new, independent admission, consistent with institutional practice. Following data cleaning, a duplicate-free dataset containing one record per patient was created as the sampling frame.

Patient records were then stratified by month of ICU admission, and a time-stratified random sampling approach, with a maximum allocation of 30 patient records per month, was applied to balance the feasibility of detailed chart review with temporal coverage across all months of the study period and to reduce the disproportionate representation of months with higher admission volumes (32). The monthly allocation was determined based on the anticipated time required for detailed manual chart abstraction within the study's available resources and personnel, consistent with established retrospective chart review methodology in which sample size and sampling strategy are frequently determined by abstraction feasibility rather than formal power calculations alone (33, 34). In months in which the number of patient records exceeded the monthly allocation, each record was assigned a computer-generated random number using Microsoft Excel. Records were sorted according to these numbers, and patients were selected sequentially until the monthly allocation was reached. In months with 30 or fewer patient records, all records were selected for eligibility screening. Screening was conducted in two stages. First, an automated search assessed the selected records against the study criteria using information from the hospital database and medication administration logs. This was followed by manual verification by a trained investigator to confirm eligibility and data completeness.

Once the final eligible patient sample had been determined, detailed data were extracted only for the selected patients. This included all antibiotic orders prescribed during each patient's admission, which were extracted from the reference file to ensure complete capture of antibiotic exposure, together with all variables specified in the standardized data collection form. Data were extracted from structured EMR modules, including admission and discharge records, laboratory and microbiology systems, and medication administration records. Unstructured documentation, such as clinical progress notes, was reviewed when clarification was required. Supplementary information on patients' clinical history during the 90 days preceding ICU admission was retrieved from Malaffi, the regional health information exchange platform.

Data collection was performed by a trained investigator using standardized operating procedures and predefined criteria based on Infectious Diseases Society of America (IDSA) and institutional guidelines to ensure consistency in data classification. To support data quality, a randomly selected subset of records was independently re-abstracted by two additional investigators. However, the exact number and proportion of records undergoing secondary review were not prospectively recorded. In addition, cases in which the primary investigator was uncertain about the classification of antibiotic appropriateness were independently assessed by the same two investigators. For this assessment, the investigators used a de-identified dataset containing only the standardized data collection fields relevant to the appropriateness evaluation. Patient identifiers and clinical outcome variables were withheld through a coded linkage maintained separately by the primary investigator. Any disagreements among the investigators were resolved through discussion and application of the same guideline-based criteria until consensus was reached. This independent review process, combined with standardized assessment criteria, was implemented to minimize bias.

### 2.6 Appropriateness of antibiotic therapy

The appropriateness of antibiotic therapy was evaluated based on adherence to the hospital's institutional antibiotic use guidelines. When relevant institutional guidelines were unavailable, recommendations from the IDSA were used as the reference standard. When uncertainty arose, the appropriateness of the antibiotic regimen was re-evaluated by two additional investigators, and disagreements were resolved through discussion and consensus between the three investigators.

The definition of appropriateness was informed by WHO and AMS frameworks and refined through a review of published studies employing comparable definitions (8, 9, 19, 29, 30). The assessment encompassed the appropriateness of the indication, empiric therapy selection, antibiotic choice, dosage and necessary dose adjustment, route and frequency of administration, duration of therapy, consideration of microbiological susceptibility results, monitoring of therapeutic drug levels (when indicated), and adherence to clinical guidelines. Antibiotic therapy was classified as inappropriate when one or more of these predefined components did not comply with the applicable recommendations. Appropriateness was assessed through a comprehensive review of the clinical information documented throughout the ICU admission, with particular consideration given to the initiation of antibiotic therapy, availability of microbiological and susceptibility results, and subsequent reassessment following therapy modification or documented changes in the patient's clinical response.

Antibiotic appropriateness was assessed at two analytical levels. At the patient level, each ICU admission was classified according to the overall appropriateness of the antibiotic therapy received during that admission. A patient was categorized as having received inappropriate antibiotic therapy if one or more components of the overall antibiotic regimen during the ICU admission were considered inappropriate according to the predefined assessment criteria. Patient-level appropriateness was calculated using the number of eligible ICU patients as the denominator (*N* = 500). At the prescription level, each individual antibiotic prescription order was evaluated separately for appropriateness. Prescription-level appropriateness was calculated using the total number of antibiotic prescriptions as the denominator (*N* = 1,605) among patients who met the study criteria and whose data were included in the analysis. The operational definitions and guidelines used during the review process are provided in Supplementary Tables S1, S2.

### 2.7 Total number of MDRO risk factors

The total number of multidrug-resistant organism (MDRO) risk factors was defined as a study-derived count variable representing the sum of predefined risk factors present during the ICU admission. Risk factor selection was informed by previous literature and published IDSA/ATS guidelines (31, 35, 36). The evaluated factors included: (1) age ≥ 65 years; (2) immunocompromised status (including patients with diabetes, chronic obstructive pulmonary disease, liver cirrhosis, uremia, or patients suffering from cancer treated with immunosuppressive drugs, radiation therapy or chemotherapy); (3) presence of a central venous catheter; (4) mechanical ventilation; (5) invasive operations such as indwelling catheter; (6) receipt of ≥3 systemic antimicrobial agents within the preceding 90 days (documented in either inpatient or outpatient settings regardless of treatment duration); (7) multiple (≥2 times) or prolonged hospitalizations within the preceding 90 days; (8) prior MDRO colonization or infection within the preceding 90 days.

Each risk factor was assigned equal weight and coded as 1 if present and 0 if absent. The total count was calculated by summing all applicable factors for each patient. When no evidence of a given risk factor was documented in the EMR or Malaffi, the factor was classified as absent.

### 2.8 Ethics approval and consent to participate

The study protocol was reviewed and approved by the Institutional Review Board of Gulf Medical University, Ajman, UAE (Reference Number: IRB-COP-STD-44-Oct-2024) and Tawam Hospital, Al Ain, UAE (Reference Number: MF2058-2025-1247), ensuring compliance with institutional data protection policies and ethical standards and prioritizing participant confidentiality and safety (Clinical trial registration: Not applicable). The study was conducted in accordance with the ethical principles outlined in the Declaration of Helsinki. Because this retrospective study involved the review of existing medical records, the requirement for informed consent was waived by the Institutional Review Board, as the research posed minimal risk to participants and did not involve direct patient contact or intervention. All data were fully anonymized and de-identified prior to analysis to ensure confidentiality. Access to patient information was restricted to authorized study investigators, and data were stored on secure, password-protected systems in accordance with institutional and national data protection regulations.

### 2.9 Statistical analysis

All statistical analyses were performed using Microsoft Excel (Professional Plus 2016) and Statistical Package for the Social Sciences (IBM SPSS Statistics for Windows, Version 22.0. IBM Corp., Armonk, NY, USA). Microsoft Excel was used for data management, including the generation of computer-generated random numbers to facilitate random patient selection, data collection, data cleaning, variable coding, and organization of the final analytical dataset. All descriptive and inferential statistical analyses were performed using IBM SPSS Statistics. Continuous variables were summarized as medians and interquartile ranges (IQRs), whereas categorical variables were expressed as frequencies and percentages. Associations between antibiotic appropriateness and categorical variables were assessed using the Chi-square test or Fisher's exact test, as appropriate. Continuous variables with non-normal distributions were compared using the Mann–Whitney *U* test. Factors independently associated with antibiotic appropriateness were identified using binary logistic regression analysis. Univariable logistic regression was initially performed, and all variables with a *p*-value ≤ 0.25 were considered for inclusion in the multivariable logistic regression model. Multicollinearity among candidate variables was assessed using variance inflation factors (VIFs) and tolerance statistics prior to finalizing the multivariable model, with a VIF < 5 and tolerance > 0.2 considered acceptable. For the analysis of clinical outcomes according to antibiotic appropriateness, binary outcomes were modeled using univariable and multivariable binary logistic regression, adjusted for the covariates independently associated with antibiotic appropriateness. Given its right-skewed distribution, ICU length of stay (LoS) was analyzed separately using a Gamma generalized linear model with an identity link through the Generalized Linear Models procedure in IBM SPSS Statistics. Crude (COR) and adjusted odds ratios (AORs) with their corresponding 95% confidence intervals (CIs) were calculated to determine the strength of association. A *p*-value < 0.05 was considered statistically significant.

## 3 Results

The initial dataset comprised 8,697 antibiotic prescription orders retrieved from the hospital database, including multiple entries per patient encounter. After consolidation of repeated orders within the same admission, 1,859 unique patient records were identified. Of these, 604 (32.5%) records were selected and screened and 104 (17.2%) were excluded because of the age criterion (*n* = 55, 52.9%), ICU LoS outside the predefined range (*n* = 30, 28.8%), antibiotic exposure outside the ICU stay (*n* = 13, 12.5%), or missing or incomplete medical records (*n* = 6, 5.8%). Consequently, 500 patients met the eligibility criteria and were included in the final analysis. The study selection process is outlined in Figure 1.

**Figure 1:**
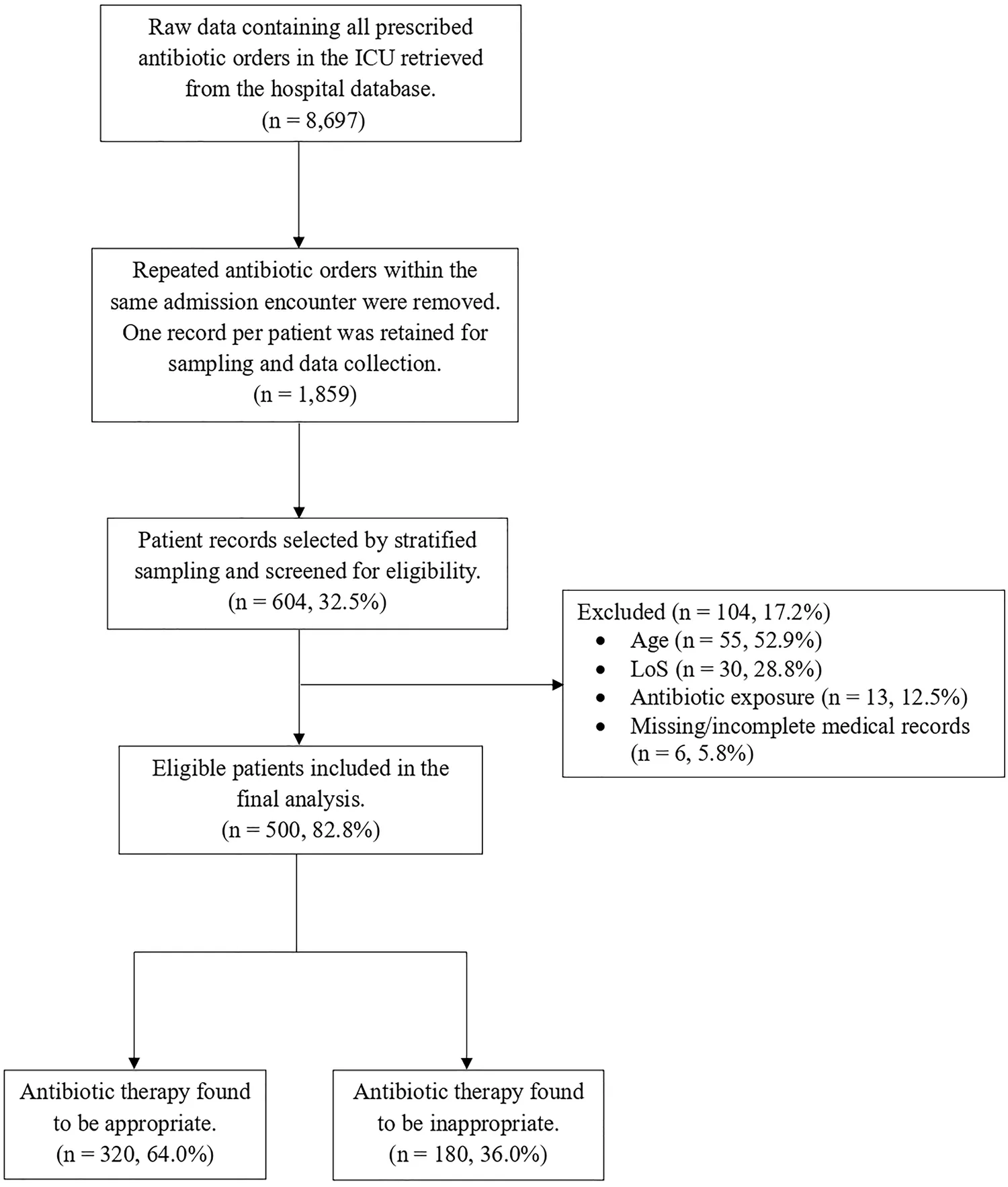
Study flow diagram of ICU patient selection.

### 3.1 Baseline characteristics of ICU patients

The baseline demographic, clinical, laboratory, and microbiological characteristics of the study cohort, stratified according to the appropriateness of antibiotic therapy, are presented in Table 1. Patients who received appropriate antibiotic therapy exhibited indicators of greater baseline illness severity and poorer prognostic profiles at ICU admission than those who received inappropriate therapy. Significant differences were observed in demographic characteristics, reasons for ICU admission, comorbidity burden, recent risk factors, ICU parameters, MDRO risk factors, and microbiological parameters. Patients in the appropriate therapy group were older, had a greater burden of comorbidities, and more frequently presented with sepsis or septic shock. They were also more likely to have a recent history of hospitalization or MDRO colonization or infection. In addition, they more frequently received enteral or parenteral nutrition, experienced organ dysfunction, had a greater number of MDRO risk factors, and had positive cultures or MDRO isolates.

**Table 1:** Baseline characteristics of ICU patients stratified according to the appropriateness of antibiotic therapy.

Variable	Total (*N* = 500)	Appropriate therapy (*n* = 320)	Inappropriate therapy (*n* = 180)	*p*-value*
Demographic characteristics				
Age, years, median (*IQR*)	47 (35–64)	52 (36–66)	42 (33–57)	<0.001
BMI, kg/m^2^, median (*IQR*)	26.3 (22.5–31.1)	26.3 (22.5–31.1)	27.1 (23.0–31.7)	0.180
ICU LoS, days, median (*IQR*)	5.5 (4–9)	6 (4–9)	5 (4–8)	0.285
*Gender*:				
Female, *n* (%)	239 (47.8)	144 (45.0)	95 (52.8)	0.095
Male, *n* (%)	261 (52.2)	176 (55.0)	85 (47.2)	
*Ethnicity*:				
UAE nationals, *n* (%)	136 (27.2)	96 (30.0)	40 (22.2)	0.057
Other Arabs^a^, *n* (%)	99 (19.8)	64 (20.0)	35 (19.4)	
Asian^b^, *n* (%)	178 (35.6)	109 (34.1)	69 (38.3)	
African^c^, *n* (%)	81 (16.2)	50 (15.6)	31 (17.2)	
Others, *n* (%)	6 (1.2)	1 (0.3)	5 (2.8)	
Reasons for ICU admission^d^				
Sepsis, *n* (%)	207 (41.4)	160 (50.0)	47 (26.1)	<0.001
Septic shock, *n* (%)	99 (19.8)	74 (23.1)	25 (13.9)	0.013
Postoperative, *n* (%)	145 (29.0)	67 (20.9)	78 (43.3)	<0.001
Trauma, *n* (%)	32 (6.4)	15 (4.7)	17 (9.4)	0.037
DKA, *n* (%)	15 (3.0)	11 (3.4)	4 (2.2)	0.445
Comorbidities				
Renal impairment^e^, *n* (%)	104 (20.8)	83 (25.9)	21 (11.7)	<0.001
Cardiovascular disorders, *n* (%)	213 (42.6)	155 (48.4)	58 (32.2)	<0.001
Neurological/neurodegenerative disorders, *n* (%)	32 (6.4)	21 (6.6)	11 (6.1)	0.843
Gastrointestinal disorders, *n* (%)	37 (7.4)	27 (8.4)	10 (5.6)	0.237
Endocrine disorders, *n* (%)	194 (38.8)	143 (44.7)	51 (28.3)	<0.001
Respiratory disorders, *n* (%)	46 (9.2)	28 (8.8)	18 (10.0)	0.643
Musculoskeletal disorders, *n* (%)	19 (3.8)	13 (4.1)	6 (3.3)	0.682
Vascular disorders, *n* (%)	44 (8.8)	31 (9.7)	13 (7.2)	0.350
Hematologic disorders, *n* (%)	36 (7.2)	26 (8.1)	10 (5.6)	0.286
Urinary disorders, *n* (%)	17 (3.4)	14 (4.4)	3 (1.7)	0.128
Cancer-related conditions, *n* (%)	112 (22.4)	69 (21.6)	43 (23.9)	0.549
Autoimmune diseases, *n* (%)	18 (3.6)	13 (4.1)	5 (2.8)	0.459
Recurrent infections, *n* (%)	21 (4.2)	16 (5.0)	5 (2.8)	0.234
Post COVID-19 syndrome, *n* (%)	2 (0.4)	2 (0.6)	0 (0)	0.538
Recent risk factors (preceding 90 days)				
Prior hospitalization history, *n* (%)	259 (51.8)	180 (56.3)	79 (43.9)	0.008
Prior invasive procedure, *n* (%)	221 (44.2)	126 (39.4)	95 (52.8)	0.005
Prior systemic antimicrobial use, *n* (%)	352 (70.4)	224 (70.0)	128 (71.1)	0.794
Prior MDRO colonization or infection, *n* (%)	41 (8.2)	34 (10.6)	7 (3.9)	0.008
ICU parameters				
Mechanical ventilation, *n* (%)	248 (49.6)	162 (50.6)	86 (47.8)	0.541
Central venous catheter, *n* (%)	37 (7.4)	18 (5.6)	19 (10.6)	0.043
Current invasive operations (such as indwelling catheter), *n* (%)	219 (43.8)	123 (38.4)	96 (53.3)	0.001
Use of vasopressors^f^, *n* (%)	157 (31.4)	102 (31.9)	55 (30.6)	0.760
Use of systemic corticosteroid therapy^g^, *n* (%)	250 (50.0)	168 (52.5)	82 (45.6)	0.136
Received enteral/parenteral nutrition, *n* (%)	294 (58.8)	201 (62.8)	93 (51.7)	0.015
Use of sedatives, *n* (%)	214 (42.8)	133 (41.6)	81 (45.0)	0.456
Organ dysfunction, *n* (%)	155 (31.0)	119 (37.2)	36 (20.0)	<0.001
*Renal replacement therapy*				
HD, *n* (%)	27 (5.4)	21 (6.6)	6 (3.3)	0.125
CRRT, *n* (%)	70 (14.0)	52 (16.3)	18 (10.0)	0.053
*MDRO Risk Factors*				
0 risk factors, *n* (%)	17 (3.4)	8 (2.5)	9 (5.0)	0.028
1–2 risk factors, *n* (%)	161 (32.2)	100 (31.3)	61 (33.9)	
3–5 risk factors, *n* (%)	296 (59.2)	189 (59.1)	107 (59.4)	
6–7 risk factors, *n* (%)	26 (5.2)	23 (7.2)	3 (1.7)	
Microbiology parameters				
Testing performed, *n* (%)	495 (99.0)	319 (99.7)	176 (97.8)	0.059
Positive cultures, *n* (%)	226 (45.2)	180 (56.3)	46 (25.6)	<0.001
MDRO isolates, *n* (%)	78 (15.6)	64 (20.0)	14 (7.8)	<0.001

All analyses presented in this table were based on the total number of eligible ICU patients (*N* = 500) as the overall denominator. Percentages in the “Appropriate therapy” and “Inappropriate therapy” columns were calculated using the respective group totals (*n* = 320 and *n* = 180). Continuous variables were compared using the Mann–Whitney *U* test, whereas categorical variables were compared using the Chi-square test or Fisher's exact test, as appropriate. BMI, body mass index; COVID-19, coronavirus disease 2019; CRRT, continuous renal replacement therapy; DKA, diabetic ketoacidosis; HD, hemodialysis; ICU, intensive care unit; IQR, interquartile range; LoS, length of stay; MDRO, multidrug-resistant organism; UAE, United Arab Emirates.

a Other Arabs included the following countries: Syria, Jordan, Palestine, Oman, Iraq, Bahrain, KSA, Lebanon, Yemen, and Qatar.

b Asia included the following countries: Philippines, India, Bangladesh, Pakistan, Nepal, Sri Lanka, Afghanistan, Indonesia, and Iran.

c Africa included the following countries: Djibouti, Rwanda, Mauritania, Uganda, Egypt, Ethiopia, Somalia, Morocco, Tunisia, Nigeria, Sahrawi, South Africa, and Comoros.

d Reasons for ICU admission were not mutually exclusive, as patients could have more than one documented reason. Only selected reasons were presented, hence frequencies and percentages may not sum up to group sample size or 100%.

e Renal impairment included all patients with acute kidney injury, any stage of chronic kidney disease, end-stage renal disease, or hemodialysis.

f Vasopressors refers to the following used: norepinephrine, epinephrine, dopamine, dobutamine, vasopressin and phenylephrine.

g Steroids refers to the following used: dexamethasone, hydrocortisone, fludrocortisone, methylprednisolone and prednisolone all in intravenous form.

* A *p*-value < 0.05 was considered statistically significant. The *p*-values were not adjusted for multiple comparisons and are presented for exploratory purposes and should not be interpreted as confirmatory inferential findings.

### 3.2 Appropriateness of antibiotic therapy

Among the 500 ICU patients included in the study, 320 (64.0%) received antibiotic therapy deemed appropriate based on clinical guideline adherence and microbiological evidence, whereas 180 (36.0%) patients received inappropriate antibiotic therapy.

#### 3.2.1 Reasons for inappropriate antibiotic use

Patterns of inappropriate antibiotic prescribing are summarized in Table 2. The most common category was a lack of indication or unjustified use (86.7%), followed by duration-related issues (35.6%), and deviations from clinical guidelines or AMS principles (33.3%). The prescription of postoperative antibiotics without an indication (44.4%), prolonged duration of therapy (26.1%), and unnecessary Methicillin-Resistant *Staphylococcus aureus* (MRSA) coverage (20.6%) were the most frequently identified reasons for inappropriate prescribing.

**Table 2:** Reasons for inappropriate antibiotic use.

Category/Reason	Frequency, *n* (%)
Lack of indication/unjustified use (156; 86.7%)	
No indication for postoperative antibiotics	80 (44.4)
No indication for MRSA coverage	37 (20.6)
No indication for antibiotic therapy	34 (18.8)
Continued MRSA-directed therapy without microbiological confirmation	4 (2.2)
Treatment of colonization rather than infection	1 (0.6)
Guideline or stewardship deviations (60; 33.3%)	
Escalation without clinical indication	34 (18.9)
Escalation despite documented resistance	2 (1.1)
Unwarranted escalation	1 (0.6)
Missed de-escalation opportunity	5 (2.8)
Not in accordance with clinical guidelines	18 (10.0)
Duration-related issues (64; 35.6%)	
Prolonged duration of therapy	47 (26.1)
Incomplete duration of therapy	13 (7.2)
No justification for early discontinuation	3 (1.7)
Therapy discontinued despite positive culture	1 (0.6)
Drug/dose/route errors (35; 19.4%)	
Omission of loading dose for vancomycin	28 (15.6)
Lack of TDM for vancomycin	1 (0.6)
Incorrect dosing frequency	2 (1.1)
Failure to switch from intravenous to oral route	4 (2.2)
Inappropriate selection/coverage (14; 7.8%)	
Inappropriate antibiotic selection	5 (2.8)
Inadequate spectrum of coverage	4 (2.2)
Resistance to prescribed antibiotic	5 (2.8)
Duplication (4; 2.2%)	
Duplicate coverage	3 (1.7)
Duplicate therapy	1 (0.6)

Percentages for both categories and individual reasons were calculated using the total number of patients who received inappropriate antibiotic therapy (*n* = 180) as the denominator. Patients may have had more than one documented reason; therefore, the percentages do not sum to 100%. Among patients who received inappropriate antibiotic therapy, 80 patients (44.4%) had one documented reason, 57 patients (31.7%) had two reasons, and 43 patients (23.9%) had three or more concurrent reasons for inappropriateness.

MRSA, Methicillin-resistant Staphylococcus aureus; TDM, Therapeutic Drug Monitoring.

Figure 2 presents the distribution of antibiotic prescribing practices across medical, surgical, and intensive care specialties. Appropriate empiric antibiotic initiation was observed in 96.7% of patients managed by surgical specialties, 95.1% of those managed by medical specialties, and 89.3% of those managed primarily by the ICU team. Despite the high rates of appropriate empiric initiation, the overall proportion of patients receiving inappropriate antibiotic therapy was higher among those managed by surgical specialties (50.8%) than among those managed primarily by the ICU team (32.1%) or medical specialties (26.6%). The distribution of inappropriate antibiotic therapy across individual specialties is provided in Supplementary Table S3.

**Figure 2:**
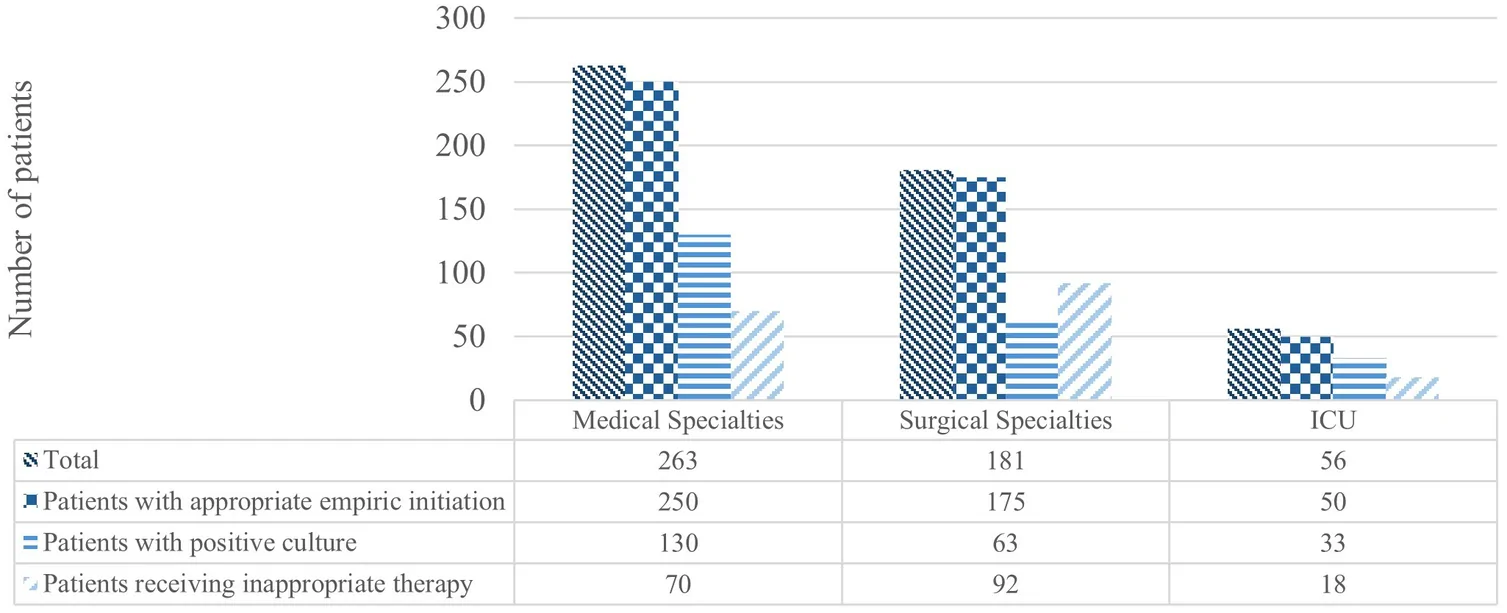
Distribution of infection-related indicators and antibiotic therapy appropriateness across medical, surgical, and ICU specialties.

#### 3.2.2 Appropriateness of frequently prescribed antibiotics

A total of 1,605 antibiotic prescription orders were recorded in the study cohort. High rates of appropriate use were observed for amikacin (96.9%), meropenem (92.2%), and piperacillin/tazobactam (87.1%). In contrast, cefepime (48.2%), metronidazole (36.2%), and cefazolin (33.9%) exhibited the highest proportions of inappropriate use. A complete breakdown of the prescribed antibiotics and their corresponding rates of inappropriate use is provided in Table 3.

**Table 3:** Complete list of prescribed antibiotics and their inappropriate use rates.

Antibiotic class	Antibiotic	ATC code	Number of prescriptions, *n* (%)^a^	Inappropriate use, *n* (%)^b^
Tetracycline	Doxycycline	J01AA02	18 (1.1)	4 (22.2)
Glycylcycline	Tigecycline	J01AA12	7 (0.4)	1 (14.3)
Penicillin	Ampicillin	J01CA01	12 (0.7)	0 (0)
	Penicillin G	J01CE01	1 (0.1)	0 (0)
	Flucloxacillin	J01CF05	5 (0.3)	1 (20.0)
Penicillin/*β*-lactamase inhibitor combinations	Co-Amoxiclav	J01CR02	19 (1.2)	4 (21.1)
	Piperacillin/tazobactam	J01CR05	295 (18.4)	38 (12.9)
Cephalosporin	Cefazolin	J01DB04	56 (3.5)	19 (33.9)
	Cefuroxime	J01DC02	17 (1.1)	13 (76.5)
	Ceftazidime	J01DD02	1 (0.1)	0 (0)
	Ceftriaxone	J01DD04	59 (3.7)	11 (18.6)
	Cefepime	J01DE01	85 (5.3)	41 (48.2)
	Ceftaroline	J01DI02	4 (0.2)	0 (0)
	Cefiderocol	J01DI04	2 (0.1)	1 (50.0)
Cephalosporin/*β*-lactamase inhibitor combinations	Ceftazidime/avibactam	J01DD52	19 (1.2)	1 (5.3)
	Ceftolozane/tazobactam	J01DI54	7 (0.4)	0 (0)
Monobactam	Aztreonam	J01DF01	16 (1.0)	1 (6.3)
Carbapenem	Meropenem	J01DH02	218 (13.6)	17 (7.8)
	Ertapenem	J01DH03	6 (0.4)	0 (0)
Sulfonamide	TMP/SMX	J01EE01	31 (1.9)	5 (16.1)
Macrolide	Clarithromycin	J01FA09	1 (0.1)	1 (100)
	Azithromycin	J01FA10	119 (7.4)	27 (22.7)
Lincosamide	Clindamycin	J01FF01	35 (2.2)	13 (37.1)
Aminoglycoside	Tobramycin	J01GB01	4 (0.2)	0 (0)
	Gentamicin	J01GB03	5 (0.3)	0 (0)
	Amikacin	J01GB06	64 (4.0)	2 (3.1)
Fluoroquinolone	Ciprofloxacin	J01MA02	9 (0.6)	0 (0)
	Levofloxacin	J01MA12	9 (0.6)	1 (11.1)
Glycopeptide	Vancomycin	J01XA01	296 (18.4)	76 (25.7)
Polymyxin	Colistin	J01XB01	1 (0.1)	0 (0)
Nitroimidazole	Metronidazole	J01XD01	69 (4.3)	25 (36.2)
Oxazolidinone	Linezolid	J01XX08	58 (3.6)	8 (13.8)
Lipopeptide	Daptomycin	J01XX09	4 (0.2)	0 (0)
Antimycobacterial agent	Ethambutol	J04AK02	13 (0.8)	0 (0)
	Isoniazid	J04AC01	14 (0.9)	0 (0)
	Pyrazinamide	J04AK01	13 (0.8)	0 (0)
	Rifampin	J04AB02	13 (0.8)	0 (0)

Each antibiotic prescription order was evaluated separately, and the analyses presented in this table were based on the total number of antibiotic prescriptions evaluated (*N* = 1,605).

ATC, anatomical therapeutic chemical; TMP/SMX, trimethoprim/sulfamethoxazole.

a Percentages in the “Number of prescriptions” column were calculated using *N* = 1,605 as the denominator.

b Percentages in the “Inappropriate use” column were calculated using the total number of prescriptions for each individual antibiotic as the denominator. Although Clarithromycin had an inappropriate-use rate of 100%, only one prescription was evaluated and therefore, this finding should be interpreted with caution.

Empiric antibiotic therapy was initiated in 475 (95.0%) patients. Culture and susceptibility testing (CST) was performed in 402 (84.6%) of these patients, and antibiotic therapy was subsequently modified in 377 (93.8%) patients based on microbiological findings. These modifications included escalation, de-escalation, optimization, and appropriate discontinuation, reflecting the widespread adoption of culture-guided antibiotic management. The antibiotic prescribing workflow is shown in Supplementary Figure S1.

### 3.3 Factors associated with antibiotic appropriateness

The results of the univariable and multivariable logistic regression analyses are shown in Table 4. In the univariable analysis, several factors were associated with antibiotic appropriateness, including age, presence of cardiovascular and endocrine diseases, renal impairment, recent hospitalization within the preceding 90 days, recent invasive surgery within the preceding 90 days, recent MDRO colonization or infection within the preceding 90 days, presence of a central venous catheter, invasive operations such as indwelling catheter, positive microbiological culture results, sepsis, and MDRO isolation during the current admission. After multivariable adjustment, three factors remained independently associated with appropriate antibiotic therapy: positive microbiological culture results (AOR = 3.34, 95% CI = 2.05–5.44, *p* < 0.001), sepsis (AOR = 2.12, 95% CI = 1.32–3.42, *p* = 0.002), and presence of a central venous catheter (AOR = 0.30, 95% CI = 0.14–0.66, *p* = 0.003). Patients with positive microbiological culture results had more than threefold higher odds of receiving appropriate antibiotic therapy, whereas those diagnosed with sepsis had approximately twofold higher odds. Conversely, the presence of a central venous catheter was associated with 70% lower odds of receiving appropriate antibiotic therapy compared with the absence of a central venous catheter. Furthermore, all VIF values were below 5, and all tolerance values were above 0.2, indicating acceptable collinearity levels among the factors.

**Table 4:** Logistic regression analysis of factors associated with antibiotic appropriateness.

Variable	COR (95% CI)	*p*-value*	AOR (95% CI)	*p*-value**
Age	1.02 (1.01–1.03)	<0.001	1.00 (0.99–1.02)	0.629
Cardiovascular disease	1.98 (1.35–2.89)	<0.001	1.04 (0.59–1.83)	0.896
Endocrine disorder	2.04 (1.38–3.02)	<0.001	1.28 (0.76–2.15)	0.357
Renal impairment	2.65 (1.58–4.46)	<0.001	1.58 (0.81–3.08)	0.182
Recurrent infections	1.84 (0.66–5.12)	0.241	0.84 (0.27–2.64)	0.769
Prior hospitalization within the preceding 90 days	1.64 (1.14–2.38)	0.008	1.04 (0.67–1.60)	0.868
Prior invasive surgery within the preceding 90 days	0.58 (0.40–0.84)	0.004	0.97 (0.41–2.31)	0.945
Prior systemic antimicrobial use within the preceding 90 days	0.95 (0.63–1.42)	0.794		
Prior MDRO colonization or infection within the preceding 90 days	2.94 (1.27–6.77)	0.011	1.51 (0.60–3.79)	0.382
Presence of central venous catheter	0.51 (0.26–0.99)	0.046	0.30 (0.14–0.66)	0.003
Mechanical ventilation	1.12 (0.78–1.62)	0.541		
Invasive operations (including indwelling catheter)	0.55 (0.38–0.79)	0.001	0.77 (0.32–1.84)	0.560
Positive microbiological culture result	3.75 (2.51–5.59)	<0.001	3.34 (2.05–5.44)	<0.001
Sepsis	2.83 (1.90–4.21)	<0.001	2.12 (1.32–3.42)	0.002
Systemic steroid use	1.32 (0.92–1.91)	0.136	0.98 (0.64–1.49)	0.916
MDRO isolation	2.96 (1.61–5.46)	<0.001	0.98 (0.46–2.07)	0.953

Logistic regression analysis was performed using the eligible ICU patient cohort (*N* = 500).

AOR, adjusted odds ratio; CI, confidence interval; COR, crude odds ratio; MDRO, multidrug-resistant organism.

* Variables with a *p*-value ≤ 0.25 in the univariable analysis were considered for inclusion in the multivariable logistic regression model.

** A *p*-value < 0.05 was considered statistically significant. The *p*-values were not adjusted for multiple comparisons and are presented for exploratory purposes and should not be interpreted as confirmatory inferential findings.

### 3.4 Association of clinical outcomes with antibiotic therapy appropriateness

Associations between antibiotic therapy appropriateness and clinical outcomes are summarized in Table 5. Patients receiving appropriate antibiotic therapy had higher rates of organ dysfunction (37.2% vs. 20.0%, *p* < 0.001), nosocomial infection (29.1% vs. 10.6%, *p* < 0.001), and persistent infection despite therapy (30.6% vs. 13.3%, *p* < 0.001). After multivariable adjustment, the associations with organ dysfunction (AOR = 1.69, 95% CI = 1.05–2.73, *p* = 0.032), nosocomial infection (AOR = 2.28, 95% CI = 1.29–4.03, *p* = 0.005) and persistent infection despite therapy (AOR = 2.13, 95% CI = 1.25–3.61, *p* = 0.005) remained significant. These residual associations may reflect incomplete adjustment for baseline illness severity and poor prognostic indicators of patients in the appropriate therapy group, as shown in the baseline characteristics, rather than a causal or harmful effect of appropriate therapy, and should be interpreted with this limitation in mind.

**Table 5:** Association between antibiotic therapy appropriateness and clinical outcomes.

Clinical outcome	Appropriate therapy, *n* (%)^a^	Inappropriate therapy, *n* (%)^a^	Total, *N* (%)^b^	COR (95% CI), p*	AOR (95% CI), p*
Mortality, *n* (%)	41 (12.8)	17 (9.4)	58 (11.6)	1.41 (0.78–2.56), *p* = 0.261	1.08 (0.57–2.07), *p* = 0.809
Organ dysfunction, *n* (%)	119 (37.2)	36 (20.0)	155 (31.0)	2.37 (1.54–3.64), *p* < 0.001	1.69 (1.05–2.73), *p* = 0.032
Nosocomial infection, *n* (%)	93 (29.1)	19 (10.6)	112 (22.4)	3.47 (2.04–5.92), *p* < 0.001	2.28 (1.29–4.03), *p* = 0.005
Persistent infection despite therapy, *n* (%)	98 (30.6)	24 (13.3)	122 (24.4)	2.87 (1.76–4.69), *p* < 0.001	2.13 (1.25–3.61), *p* = 0.005
Adverse drug events, *n* (%)	31 (9.7)	19 (10.6)	50 (10.0)	0.91 (0.50–1.66), *p* = 0.756	0.61 (0.31–1.16), *p* = 0.132
ICU LoS, days^c^	6.0 (4.0–9.0)	5.0 (4.0–8.3)	5.5 (4.0–9.0)	0.43 (−0.38 to 1.24), *p* = 0.300	−0.64 (−1.44 to 0.17), *p* = 0.120

COR was derived from unadjusted univariable logistic regression with antibiotic appropriateness as the sole predictor. AOR was derived from multivariable logistic regression adjusted for presence of central venous catheter, sepsis, and positive microbiological culture result (covariates independently associated with antibiotic appropriateness in the multivariable model). AOR, Adjusted Odds Ratio; CI, Confidence Interval; COR, Crude Odds Ratio; LoS, Length of Stay.

a Percentages in the “Appropriate therapy” and “Inappropriate therapy” columns were calculated using the respective group totals (*n* = 320 and *n* = 180) as denominators.

b Percentages in the “Total” column were calculated using the overall cohort (*N* = 500) as the denominator.

c For LoS, values represent the crude and adjusted mean difference in days from Gamma generalized linear model, given its right-skewed distribution.

*A *p*-value < 0.05 was considered statistically significant. The *p*-values are presented for exploratory purposes and should not be interpreted as confirmatory inferential findings.

## 4 Discussion

In this cohort, 64% of critically ill patients received antibiotic therapy that was deemed appropriate. This rate is comparable to findings from South Africa, where an appropriateness rate of 70.2% was reported (20). In contrast, substantially lower rates have been documented in studies from Pakistan (14%) (18) and Sudan (28%) (37), where the absence of in-house infection management guidelines and ASPs was identified as a key contributing factor. The relatively high appropriateness rate observed in our study may reflect the influence of a national ASP framework (38, 39), the presence of an active institutional ASP committee aligned with IDSA guidelines, and the integration of clinical pharmacists into the ICU multidisciplinary team. These factors have previously been associated with improved guideline adherence and reduced antimicrobial misuse (40–42).

The leading driver of inappropriate antibiotic use, identified in more than three-quarters of cases, was a lack of indication or unjustified therapy, with postoperative antibiotic use without an indication accounting for nearly half of all inappropriate prescriptions. Duration-related issues, particularly prolonged therapy (26.1%), were also frequently observed. Similar findings have been reported in Türkiye, where one study identified unnecessary antibiotic use in 38.9% of patients and prolonged surgical prophylaxis in 34.1% (10), whereas another reported a lack of indication in 47% and prolonged therapy in 26% of cases (9). Conversely, a Brazilian study highlighted different contributors to inappropriate use. Among empiric treatments, the most frequently reported reasons for inappropriateness were failure to obtain cultures and bacterial resistance (80.2%), whereas resistance to the selected antibiotics and excessive cost predominated among susceptibility-guided treatments (58.3%) (19). These variations likely reflect differences in case mix, admission profiles, local resistance epidemiology, and institutional prescribing practices across study settings. In our cohort, the relatively high proportion of postoperative ICU admissions (29%), routine continuation of surgical prophylaxis beyond 24 h, and reliance on empiric therapy may have contributed to the predominance of indication- and duration-related inappropriate use. By contrast, settings with a greater proportion of medical ICU admissions, greater microbiological complexity, or different stewardship structures may exhibit culture- or resistance-driven patterns of inappropriate use. Robust evidence indicates that extending postoperative antibiotic prophylaxis beyond 24 h offers no additional clinical benefit (43–46). Duration-related problems were also observed among nonsurgical patients, reinforcing previous evidence that shorter antibiotic courses can reduce AMR and nosocomial infections (47), while allowing treatment to be individualized based on clinical and laboratory assessments (38, 48).

Although intensivists were primarily responsible for managing ICU patients, antibiotic prescribing involved multiple medical and surgical specialties. Surgical services exhibited the highest rates of inappropriate antibiotic use, ranging from 62.5% to 72.7% across specialties. Comparable findings have been reported in Türkiye, where inappropriate prescribing was more frequent in surgical ICUs than in medical ICUs (51.4% vs. 30.7%, *p* < 0.001) (9). In contrast, a South African study reported high antibiotic overuse among cardiology patients, with 78.5% lacking a documented indication (20). These discrepancies may stem from interacting factors, including differences in admission profiles, specialty-specific prescribing cultures, case mix, institutional oversight mechanisms, and the availability of stewardship resources, such as infectious diseases consultation and clinical pharmacist involvement. In our setting, the combination of a substantial surgical ICU burden, variation in perioperative prescribing practices across specialties, and limited structured reassessment of empiric therapy may have contributed to the higher rates of inappropriate use among surgical services, particularly regarding cefepime. The high rate of inappropriate cefepime prescribing (48.2%) appears to be largely driven by its postoperative use beyond the recommended prophylactic indications or duration. This finding may reflect gaps in AMS implementation within surgical services and institutional prescribing practices, including the routine continuation of prophylactic antibiotics without a clear clinical indication. Such continuation may be driven by concerns regarding postoperative infections rather than adherence to guideline-recommended durations. These findings emphasize the need to strengthen specialty-specific stewardship interventions, particularly within surgical departments, through regular audits, targeted feedback, and multidisciplinary engagement, which have been shown to improve prescribing practices (28). Additional measures, such as standardized perioperative antibiotic protocols, automatic stop orders, and closer involvement of the AMS team in surgical care, may further reduce unnecessary antibiotic exposure.

Among the antibiotic classes, cephalosporins demonstrated some of the highest rates of inappropriate use, particularly cefepime (48.2%) and cefazolin (33.9%). Cefuroxime and cefiderocol also had high rates of inappropriate use at 76.5% and 50.0%, respectively; however, these estimates should be interpreted cautiously because of the small number of prescriptions. These patterns were especially evident among surgical specialties, suggesting suboptimal prophylactic prescribing practices within these departments. Comparable trends have been reported in Kenya, where inappropriate use was documented for cefuroxime (93.3%), ceftriaxone (83%), and cefazolin (85.7%) (5). This pattern is concerning given the intrinsic resistance of organisms such as *Pseudomonas aeruginosa* and *Enterococcus* species to first- and second-generation cephalosporins (49). Furthermore, the UAE National AMR Surveillance Report (2023) documented rising resistance to third- and fourth-generation cephalosporins among *Escherichia coli* and *Klebsiella pneumoniae* (25).

The CST plays a critical role in supporting appropriate antimicrobial decision-making (43, 44). In this study, empiric therapy was initiated in 95% of patients, CST was performed in 84.6%, and antibiotic therapy was subsequently modified in 93.8% of those tested. In contrast, a South African study reported lower CST rates (61.2%) and de-escalation (13.1%) (20), while a Sudanese study reported empiric antibiotic initiation in 80.4% of patients but CST performance and therapy modification in only 18.6% and 8.8%, respectively (8). These findings suggest that timely microbiological testing and the subsequent interpretation of results were well integrated into the stewardship process in our setting. This observation was further supported by the multivariable logistic regression analysis, which identified a positive microbiological culture result as a factor independently associated with appropriate antibiotic therapy (AOR = 3.34, 95% CI = 2.05–5.44, *p* < 0.001). This finding is consistent with a systematic review and meta-analysis by Raman et al., which demonstrated significantly lower treatment failure when antimicrobial therapy was guided by microbiological data (OR 0.22, 95% CI 0.14–0.35) (50). Similarly, a study from Singapore evaluating culture status among patients with severe sepsis reported that patients with positive cultures were more likely to receive tailored and optimized therapy after the results became available (11). Collectively, these findings support the role of timely pathogen identification and effective interpretation of microbiological findings in improving antibiotic appropriateness and reinforcing culture-guided prescribing practices. In addition to positive microbiological culture results, the multivariable logistic regression analysis identified sepsis and the presence of a central venous catheter as other factors independently associated with appropriate antibiotic therapy. Although these associations were statistically significant, their effect sizes varied and should be interpreted within the broader clinical context of antibiotic prescribing in critically ill patients.

Sepsis was associated with higher odds of receiving appropriate antibiotic therapy (AOR = 2.12, 95% CI = 1.32–3.42, *p* = 0.002). This finding aligns with a systematic review by Paul et al., which reported improved survival among patients who received appropriate antimicrobial therapy within the first 48 h of sepsis management (95% CI = 1.37–1.86) (51). A study from Indonesia further demonstrated a significant association between inappropriate antibiotic dosing and higher mortality among patients with sepsis (*p* = 0.034) (52). The higher likelihood of appropriate therapy among patients with sepsis may reflect the urgency of early antimicrobial administration emphasized in the Surviving Sepsis Campaign guidelines, which recommend the prompt initiation of empiric broad-spectrum antibiotics, followed by reassessment and optimization as clinical and microbiological information becomes available (30, 53).

Patients with a central venous catheter had around 70% lower odds of receiving appropriate therapy than those without a central venous catheter (AOR = 0.30, 95% CI = 0.14–0.66, *p* = 0.003). This association may reflect the complexity of infections and greater illness severity among patients requiring invasive devices, in whom device-related complications may influence antibiotic selection and increase the likelihood of deviation from guideline-recommended therapy. Additionally, the presence of a central venous catheter may serve as a proxy for a prolonged ICU stay, greater exposure to broad-spectrum antibiotics, and an increased risk of healthcare-associated infections. Yokota et al. reported that although 81% of patients in their study received appropriate antibiotic therapy, none of the assessed variables were significantly associated with inappropriate therapy among patients with central line-associated bloodstream infections (54). Evidence examining the relationship between central venous catheter use and antibiotic appropriateness remains limited. Most existing studies have focused primarily on the incidence, prevention, or mortality associated with central line-related infections rather than on the appropriateness of antibiotic therapy throughout the treatment course, thereby limiting direct comparisons with the present findings.

Analysis of clinical outcomes revealed that patients receiving appropriate antibiotic therapy had higher crude rates of organ dysfunction (37.2% vs. 20.0%, *p* < 0.001), nosocomial infection (29.1% vs. 10.6%, *p* < 0.001), and persistent infection (30.6% vs. 13.3%, *p* < 0.001). After adjustment for positive microbiological culture result, sepsis, and presence of central venous catheter, these associations remained significant for organ dysfunction (AOR = 1.69, 95% CI = 1.05–2.73, *p* = 0.032), nosocomial infection (AOR = 2.28, 95% CI = 1.29–4.03, *p* = 0.005) and persistent infection despite therapy (AOR = 2.13, 95% CI = 1.25–3.61, *p* = 0.005). These findings should be interpreted cautiously, as patients receiving appropriate therapy also demonstrated several indicators of greater baseline illness severity, including higher rates of sepsis, positive microbiological cultures, and multidrug-resistant organisms. This pattern likely reflects confounding by indication, where patients with clearer or more severe infectious presentations are more likely to receive guideline-concordant therapy. Consequently, the higher complication rates observed in this group should not be interpreted as evidence that appropriate antimicrobial therapy worsens outcomes. In contrast, several studies have reported increased infection persistence, higher mortality (8, 18), and prolonged ICU stay among patients receiving inappropriate antibiotic therapy (19, 31). Differences across studies may reflect variations in patient case mix, severity of illness, infection burden, and AMS practices. In critically ill populations, patients who survive longer or present with more severe infections may remain at risk of nosocomial complications due to prolonged ICU exposure, invasive devices, and hospital flora. In addition, effective ASPs and infection control practices may optimize antimicrobial therapy and support improved survival even among patients with severe disease.

This study has several limitations. First, its retrospective design relied on the accuracy and completeness of EMRs, and incomplete documentation occasionally limited data extraction, resulting in the exclusion of some patients. As this was a single-center study, the generalizability of the findings may be limited. The use of time-stratified random sampling may have introduced selection bias by limiting the number of included cases from months with high admission volumes while including all eligible cases from months with lower admission volumes. This approach may have affected the representativeness of the study population and reduced the precision and statistical power of estimates involving less common exposures or outcomes. Although random selection was applied within higher volume months to mitigate selection bias, the possibility of residual bias cannot be excluded. Sensitivity analyses were not performed because access to excluded records was not feasible, thereby limiting the assessment of robustness and stability of the findings under alternative assumptions. In addition, the presence of multiple concurrent diagnoses in some patients made the assessment of antibiotic appropriateness challenging before infection was confirmed. The limited availability of institution-specific antibiotic guidelines necessitated reliance on IDSA guidelines, which, although standardized, may not fully reflect local AMR patterns, formulary restrictions, or institution-specific prescribing practices. Although a predefined secondary review procedure was used to support consistency in data extraction and clinical judgment, the exact number and proportion of records independently reviewed were not prospectively recorded. Formal inter-rater agreement could therefore not be quantified, which may limit the reproducibility of the assessment. Furthermore, the absence of standardized ICU severity scores (APACHE II or SOFA) limited adjustment for baseline illness severity, including through advanced causal inference methods such as propensity score matching or inverse probability weighting. Consequently, the observed associations between antibiotic appropriateness and clinical outcomes may have been influenced by residual confounding and confounding by indication, and causal inferences cannot be established. In addition, although logistic regression was used, the relatively high outcome prevalence may have resulted in modest overestimation of effect sizes when expressed as odds ratios.

Several real-world systemic and operational barriers may also have influenced antibiotic prescribing. The limited availability of infectious diseases consultants restricted consistent follow-up and timely reassessment of ICU cases, particularly those requiring rapid treatment escalation for MDROs. Communication gaps and suboptimal multidisciplinary integration may have resulted in overlapping or conflicting antibiotic-related decisions. Inconsistent adherence to available guidelines and limited awareness of local resistance patterns may have persisted among prescribers, despite the presence of an established ASP. Inadequate clinical documentation further constrained real-time stewardship activities. Finally, defensive prescribing among clinically deteriorating patients may have contributed to the unnecessary continuation of broad-spectrum empiric therapy.

## 5 Conclusion

Approximately one-third of critically ill patients received inappropriate antibiotic therapy, representing a substantial stewardship concern. Persistent challenges, such as unjustified postoperative antibiotic use and prolonged treatment durations, remained evident despite the presence of an established ASP. Antibiotic overuse was particularly evident within certain surgical specialties.

## 6 Recommendations

To enhance AMS effectiveness, interventions must extend beyond traditional stewardship models. Key strategies include strengthening interprofessional communication, clearly defining stewardship roles across subspecialties, enforcing thorough documentation of antibiotic indications, reviewing treatment timelines and stop dates (e.g., 72-hour time-out), providing continuing education for physicians and residents, reinforcing the role of clinical pharmacists through bedside stewardship audits, and ensuring timely infectious diseases consultations and follow-up. Routine evaluation of stewardship program performance and implementation of tailored strategies to improve prescriber adherence to guidelines and responsiveness to local resistance patterns are also essential. Collectively, these measures may help reduce inappropriate antibiotic use and improve AMS practices among critically ill patients in the ICU.

## 7 Future directions

Future multicenter prospective studies incorporating standardized severity scoring systems, longitudinal follow-up, and advanced causal inference methods are needed to better characterize the relationship between antibiotic appropriateness and clinical outcomes in critically ill populations. Such studies may also help validate factors associated with inappropriate antibiotic use and identify patient subgroups at greatest risk.

Beyond these methodological priorities, future research should evaluate the comparative effectiveness of specialty tailored AMS strategies in reducing the inappropriate continuation of postoperative antibiotics in surgical ICUs. Their effects on prescribing appropriateness and clinical outcomes should be examined across diverse institutional settings.

## Data Availability

The raw data supporting the conclusions of this article will be made available by the authors, without undue reservation.
