# Supplementary material for: Antibiotic prescribing appropriateness among critically ill patients at a single tertiary care center in the United Arab Emirates: a retrospective cohort study
Source: Front Health Serv. 2026 Sep 30;6:1900610. doi: 10.3389/frhs.2026.1900610 (PMC13662006; doi:10.3389/frhs.2026.1900610)
Supplement: Supplementary file 1 [file Datasheet1.docx]

**Supplementary Appendix**

Table S1. Operational definitions

| **Term** | **Operational definition** | **Example** |
| --- | --- | --- |
| Inappropriate antibiotic selection | Selection of an antimicrobial agent that does not align with accepted guidelines, or available microbiological results despite the availability of appropriate alternatives.  This includes situations where the antibiotic is:   - Not indicated for the diagnosis - Does not follow guideline or practice - Excessively broad without justification - Ineffective based on known resistance pattern | Adding Azithromycin to the treatment regimen of hospital-acquired pneumonia (HAP). |
| Inadequate spectrum of coverage | Use of an antimicrobial agent that fails to cover one or more likely or confirmed pathogen. This is based on:   - Infection source - Patient risk factors - Microbiological data | Intra-abdominal sepsis treated with Ceftriaxone alone, when a more proper spectrum that covers anaerobes should have been initiated. (ex: Metronidazole or Piperacillin/Tazobactam) |

***Table S2.*** *In-house and international guidelines for infection management and antimicrobial therapy review*

**Guidelines:**

All of the following guidelines were used in reviewing infection and treatment therapies.

**In-house available guidelines:**

1. Management of Febrile Neutropenia in Adult Cancer patients, 2023
2. Empirical Treatment of Adult Urinary Tract infection, 2024
3. Adult Intravenous Vancomycin Dosing and Monitoring Guideline, 2024
4. IV Vancomycin Dosing Guideline in Adult Patients, 2018
5. IV Colistin Dosing Protocol in Adult Patients, 2024
6. Surgical prophylaxis guideline, 2019

***Table S2a.*** *In-house guidelines for infection management and antimicrobial therapy review*

| **Guideline** | **Empirical therapy**  **(initial management)** | **Alternative treatment/Directed therapy** | **Remarks** |
| --- | --- | --- | --- |
| **In-house hospital guidelines** | | | |
| **Management of Febrile Neutropenia in Adult Cancer patients, 2023** | -Piperacilin/Tazobactam 4.5 g IV every 6 hours.  **OR**  -Cefepime 2 g IV every 8 hours *(type 2 allergy-non anaphylactic hypersensitivity)*.  **PLUS**  -Amikacin 15mg/kg IV once daily x 3 days (review culture in 24-48 hrs and discontinue if not indicated).  **OR**  -Meropenem *(if colonization with MDRO infections, high suspicion of ESBL infections or severe sepsis)*1g every 8 hours.  **Add**  -Vancomycin Loading dose 25mg/kg then 15mg/kg IV every 12-8 hours. | In case of serious documented beta-lactam allergy.  -Aztreonam 2 g IV every 8 hours.  **AND**  -Vancomycin LD 25mg/kg then 15mg/kg every 12-8h.  **AND**  -Amikacin 15mg/kg once daily x 3 days 3 review culture in 24 to 48 hrs and discontinue if not indicated. | **In case of positive culture:**  **•** Adjust Antibiotics according to cultures. *(if applicable)*  **•** Continue antibiotics to complete the course of therapy.  **•** Monitor for resolution of infection.  **•** Consider stepping down antibiotic and change from IV to oral.  **In case of negative culture:**  **•** Discontinue antibiotics once ANC > 500 OR after 5 days if patient is afebrile for 48hrs and ANC was 500.  **•** Reassess if spiked fever again. |
| **Empirical Treatment of Adult Urinary Tract infection, 2024** | **Uncomplicated UTI and simple Cystitis** | | Broad spectrum antibiotics are indicated in patients with urosepsis and those with risk factors for an MDR gram negative infection if:  • There is recent use of antibiotics *(within 3 months)*  • There is an MDR gram negative uropathogens *(within 3 months)*  If there is recent hospitalization *(within 3 months)*  • Piperacillin/tazobactam 4g IV every 6 hours  • Ertapenem 1g every 24 hours  • Meropenem 1g every 8 hours |
|  | -Nitrofurantoin 50mg-100mg PO every 6 hours for 5 days.  **OR**  -Fosfomycin 3g PO once daily for 3 days.  **OR**  -Amikacin 15mg/kg or gentamicin 5mg/kg once.  **OR**  -Co-Amoxiclav 875 mg PO every 12 hours for 5-7 days. | -Trimethoprim-Sulfamethoxazole 160/800 mg PO every 12 hours for 3 days.  **OR**  -Trimethoprim 100 mg PO every 12 hours for 3 days.  **OR**  -Cefuroxime 500 mg PO every 12 hours for 5-7 days.  **OR**  -Fluoroquinolones (Ciprofloxacin 500mg, or Levofloxacin 500 mg) PO for 3 days. |  |
|  | **Acute complicated UTI and Pyelonephritis** | |  |
|  | -Cefoxitin 2g every 6 hours IV for 10-14 days.  **OR**  -Gentamicin 5mg/kg every 24 hours or Amikacin 15 mg/kg IV for 3 days. | -Trimethoprim-Sulfamethoxazole 160/800 mg PO every 12 hours for 10-14 days.  **OR**  -Co-Amoxiclav 875 mg PO every 12 hours/1g every 8 hours IV for 10-14 days.  **OR**  -Fluoroquinolones (Ciprofloxacin 500mg every 12 hours, or Levofloxacin 500 mg every 24 hours for 7-10 days.) |  |
|  | **UTIs in pregnancy & Asymptomatic bacteriuria** | | **Treat asymptomatic bacteriuria only in:**  • Pregnancy  • Patients going for urological procedure where mucosal injury is expected.  **•** Patients with a positive urine culture and/or pyuria and have no UTI signs or symptoms should not be treated with antibiotics.  **•** Do not treat asymptomatic candiduria.  **•** Do not treat asymptomatic catheter related UTIs. |
|  | -Cefuroxime 500 mg PO every 12 hours for 4-7 days.  **OR**  -Amoxicillin 500 mg PO every 8 hours or 875 mg PO every 12 hours for 4-7 days.  **OR**  -Co-Amoxiclav 875 mg PO every 12 hours for 4-7 days.  **OR**  -Fosfomycin 3g PO once daily for 3 days. |  |  |
| **Adult Intravenous Vancomycin Dosing and Monitoring Guideline, 2018, 2024** | **Loading dose:**  A loading dose of 25 mg/kg *(based on actual body weight, maximum of 3g/dose)* may be considered to rapidly achieve target concentrations.  Loading doses in obese patients *(BMI⩾30, AKI, CrCl <30 mL/min can be given 20mg/kg based on actual body weight.)*  **Maintenance dose:**  A maintenance dose of 15 mg/kg every 12-8 hours. *(based on actual body weight)* with the maximum of 2g/dose and 4.5g per 24 hours initially *(including loading dose that has been administered)* |  | • Dose adjustments should be guided by vancomycin troughs *(target 15 to 20 mg/L for severe infections, 10-15 mg/L for other infections including e.g. skin and soft tissue infections; purulent cellulitis)*  • Trough levels are monitored 30 min prior to 4th dose in normal kidney function. The next dose should be given at the scheduled time. Do not wait for the trough level as the 5th dose will be adjusted according to the trough level.  • Trough levels are monitored 30 min prior to 3rd dose in kidney impairment/ AKI. Withhold next dose until level is known.  • If target trough level is achieved at steady state in patients with normal kidney function, trough levels should be checked once weekly along with kidney function tests until completion of therapy or discharge.  • Trough level and Scr should be checked sooner when clinically warranted *(e.g.*  *change in clinical status or unstable kidney function, concern of accumulation,*  *concomitant nephrotoxic medications)* |
| **IV Colistin Dosing Protocol in Adult Patients, 2024** | **Loading dose:**  9 million units (MU) to all patients with normal and impaired renal function *(Hemodialysis, Continuous Renal Replacement Therapy)*.  **Maintenance dose:**  Start daily maintenance dosing 12 hours after loading dose. The dosing recommendations should achieve a steady-state of 2 mg/L of colistin plasma concentration.  CrCl to dosing frequency:  -80 mL/min >> 5 MU every 12 hours.  - 50-79 mL/min >> 4 MU every 12 hours.  - 30-49 mL/min >> 3 MU every 12 hours.  - 10-29 mL/min >> 2.5 MU every 12 hours.  - <10 mL/min >> 2 MU every 12 hours. |  | • Use adjusted body weight when calculating CrCL.  • Use Ideal body weight in obese patients BMI > 30 kg/m2 when calculating CrCL.  • Once daily dosing is not recommended due to potential for toxicity and lack of efficacy data.  • IV administration may not be adequate for the treatment of lung infections in critically ill  patients, especially those caused by organisms that have elevated MIC organisms.  **• In case of HD;**  • Non-dialysis days>> 2 MU every 12 hours **AND** 2 MU every 12 hours plus a supplemental dose of 1 MU post-HD with the next regular dose.  **• In case of CRRT;** 5 MU every 12 hours. |
| **Surgical prophylaxis guideline, 2019**  **Surgical prophylaxis guideline, 2019**  ***(continued)***    **Surgical prophylaxis guideline, 2019**  ***(continued)*** | **Thoracic surgery** | | |
|  | - Cefazolin 2g | -Cefuroxime 1.5g  **In case of penicillin allergy:**  -Vancomycin 15 mg/kg | **•** Note on endocarditis prophylaxis: Patients receiving pre-op antibiotics to prevent an SSI generally **do NOT** need additional antibiotics for endocarditis prophylaxis. |
|  | **Vascular surgery** | | |
|  | - Cefazolin 2g | -Cefuroxime 1.5g  **In case of penicillin allergy:**  -Vancomycin 15 mg/kg |  |
|  | **Esophagus-Gastroduodenal** | | |
|  | - Cefazolin 2g | -Cefuroxime 1.5g  **In case of penicillin allergy:**  -Clindamycin 900 mg **AND** Gentamicin 5mg/kg | **• High risk only:** morbid obesity, esophageal obstruction, decreased gastric acidity or gastrointestinal motility |
|  | **Biliary tract** | | |
|  | - Cefazolin 2g | -Cefuroxime 1.5g  **In case of penicillin allergy:**  -Clindamycin 900 mg **AND** Gentamicin 5mg/kg | **• High risk only:** age>70 years, acute cholecystitis, non-functioning gallbladder, obstructive jaundice or common duct stones |
|  | **Non-obstructed small intestine surgery** | | |
|  | - Cefazolin 2g | -Cefuroxime 1.5g  **In case of penicillin allergy:**  -Clindamycin 900 mg **AND** Gentamicin 5mg/kg |  |
|  | **Obstructed small intestine surgery** | | |
|  | -Cefazolin 2g **AND** Metronidazole 500 mg | -Co-Amoxiclav 2.4 g  **In case of penicillin allergy:**  -Metronidazole 500 mg AND Gentamicin 5mg/kg |  |
|  | **Colorectal surgery** | | |
|  | -Cefazolin 2g **AND** Metronidazole 500 mg | -Co-Amoxiclav 2.4 g  **OR**  -Neomycin sulfate 1g **AND**  **-**Erythromycin 1g at 19-, 18- and 9-hours prior surgery  **In case of penicillin allergy:**  -Clindamycin 900 mg **AND** Gentamicin 5mg/kg |  |
|  | **Non-perforated appendectomy** | | |
|  | -Cefazolin 2g **AND** Metronidazole 500 mg | Co-Amoxiclav 2.4 g  **In case of penicillin allergy:**  -Clindamycin 900 mg **AND** Gentamicin 5mg/kg | **•** If perforation has occurred, antibiotics are often continued for 5 to 7 days. |
|  | **Head and Neck surgery** | | |
|  | -Clindamycin 900 mg **AND** Gentamicin 5mg/kg | -Cefazolin 2g **AND** Metronidazole 500 mg  **In case of penicillin allergy:**  -Clindamycin 900 mg |  |
|  | **Neurological procedures** | | |
|  | -Cefazolin 2g | -Clindamycin 900 mg  **In case of penicillin allergy:**  -Vancomycin 15 mg/kg |  |
|  | **Vaginal, Abdominal or Laparoscopic Hysterectomy** | | |
|  | -Cefazolin 2g | -Co-Amoxiclav 2.4g  **In case of penicillin allergy:**  -Clindamycin 900 mg **AND**  **-**Gentamicin 5mg/kg |  |
|  | **Cesarean Delivery** | | |
|  | -Cefazolin 2g | -Co-Amoxiclav 2.4g  **In case of penicillin allergy:**  -Clindamycin 900 mg | **•** Prophylaxis should be given within one hour before incision. |
|  | **Orthopedic Surgery** | | |
|  | -Cefazolin 2g | -Cefazolin 2g  **In case of penicillin allergy:**  -Vancomycin 15 mg/kg | **•** Patients with orthopedic hardware also do not appear to need additional prophylaxis beyond what is recommended for the case.  **•** There are no recommendations for antibiotics for these patients in cases where they are not indicated for SSI prevention. |
|  | **Plastic Surgery** | | |
|  | -Cefazolin 2g | -Cefuroxime 1.5g  **In case of penicillin allergy:**  -Clindamycin 900 mg |  |

**International guidelines:**

1. 2024 Global Initiative for Chronic Obstructive Lung Disease
2. AAOS Prevention of surgical site infections after major extremity trauma
3. American college of gastroenterology guidelines management of acute pancreatitis
4. Diagnosis and Management of Complicated Intra-abdominal Infection in Adults and Children Guidelines by the Surgical Infection Society and the Infectious Diseases Society of America
5. IDSA 2016 Clinical Practice Guidelines for the Management of Adults with Hospital-acquired and Ventilator-associated Pneumonia
6. IDSA 2017 Clinical Practice Guidelines for Healthcare-Associated Ventriculitis and Meningitis
7. IDSA Clinical Practice Guidelines for the Diagnosis and Management of Skin and Soft Tissue Infections 2014 Update
8. IDSA Guidelines for Diagnosis and Treatment of Adults with Community-acquired Pneumonia
9. IDSA Guidelines for the Management of Encephalitis
10. IDSA Guidelines on the Diagnosis and Treatment of Diabetes-related Foot Infections
11. IDSA Guidelines on The Practice of Travel Medicine
12. IDSA Practice Guidelines for the Management of Bacterial Meningitis
13. IDSA Updates on the Treatment of Drug-Susceptible and Drug-Resistant Tuberculosis
14. Infectious Diseases Society of America 2024 Guidance on the Treatment of Antimicrobial-Resistant Gram-Negative Infections Infectious
15. SIS IDSA Guidelines on Diagnosing and Managing Complicated Intra-Abdominal Infections in Adults and Children 2016
16. Surviving Sepsis Campaign: International Guidelines for Management of Sepsis and Septic Shock 2021

***Table S2b.*** *International guidelines for infection management and antimicrobial therapy review*

| **Guideline** | **Empirical therapy**  **(initial management)** | **Alternative treatment/Directed therapy** | **Remarks** |
| --- | --- | --- | --- |
| **International guidelines** | | | |
| **2024 Global Initiative for Chronic Obstructive Lung Disease** | -Azithromycin (250 mg/day or 500 mg three times daily per week)  **OR**  -Erythromycin (250 mg every 12 hours per day) for one year for patients prone to exacerbations. | **Pulse therapy:**  Moxifloxacin (400 mg/day for 5 days every 8 weeks) in patients with chronic bronchitis and frequent exacerbations. |  |
| **AAOS Prevention of surgical site infections after major extremity trauma** | -Cefazolin 2g  **OR**  -Clindamycin 900 mg | **For Type III fractures:**  -Piperacillin/Tazobactam 4.5 g | **•** In patients with major extremity trauma undergoing surgery, it is  recommended that antibiotic prophylaxis be administered, except for Type III (and possibly Type II) open fractures, for which additional Gram-negative coverage is preferred.  **•** In closed and open fractures (except Type III and possibly Type II open fractures),  there is no need to continue antibiotic prophylaxis longer than a day. |
| **American college of gastroenterology guidelines management of acute pancreatitis** | **In case of infected pancreatic necrosis:** | | |
|  | -Carbapenems (Imipenem-Cilastatin 500 mg IV every 6 hours, or 1g every 8 hours)  **OR**  -Meropenem 1g IV every 8 hours | -Piperacillin-Tazobactam 4.5g IV every 6-8 hours  **OR**  -Ciprofloxacin 400 mg IV every 12 hours  **AND** Metronidazole 500 mg IV every 8 hours  **OR**  -Levofloxacin 750 mg IV every 24 hours  **AND** Metronidazole 500 mg IV every 8 hours | **•** Do not use prophylactic antibiotics in sterile necrosis or mild pancreatitis. Always adjust dosing according to renal functions.  **•** Duration: 2-3 weeks for infected necrosis based on source control and patient improvement.  **•** Patient can be stepped down to oral Ciprofloxacin and Metronidazole only after clinical stabilization. |
|  | **Suspected cholangitis or biliary source** | | |
|  | -Piperacillin-Tazobactam 4.5g IV every 6-8 hours  **OR**  Carbapenems | Same as above | Adjust accordingly after cultures and ERCP. |
| **Diagnosis and Management of Complicated Intra-abdominal Infection in Adults and Children Guidelines by the Surgical Infection Society and the Infectious Diseases Society of America** | **Mild-to-moderate community acquired infection** | | |
|  | **Single-agent therapy:**  -Cefoxitin 2g IV every 6 hours  **OR**  -Ertapenem 1g IV every 24 hours  **OR**  -Moxifloxacin 400 mg IV/PO every 24 hours  **OR**  -Tigecycline 100 mg IV loading dose > 50 mg every 12 hours  **Combination therapy:**  -Cefazolin 2g IV every 24 hour  **OR**  **-**Cefuroxime 1.5g IV  **OR**  **-**Ceftriaxone 1-2g IV every 24 hours  **OR**  **-**Cefotaxime1-2g IV every 6-8 hours  **AND** Metronidazole 500 mg IV every 8 hours.  **In case of Beta-lactam allergy:**  -Ciprofloxacin 400 mg IV every 12 hours  **OR**  **-**Levofloxacin 750 mg IV every 24 hours  **AND** Metronidazole 500 mg IV every 8 hours. | | **This includes:**  **•** Perforated or abscessed appendicitis  **•** Other infections of  mild-to-moderate severity  **•** Bowel injuries attributable to penetrating, blunt, or iatrogenic trauma that are repaired within 12 hours and any other  intraoperative contamination of the operative field by enteric  contents should be treated with antibiotics for <24 hours. |
|  | **High-Risk or Severe Community-Acquired infection/ Healthcare infections of any severity** | | |
|  | **Single-agent therapy:**  -Piperacillin/Tazobactam 3.375-4.5g IV every 6 hours  **OR**  -Meropenem 1g IV every 8 hours  **OR**  -Imipenem-Cilastatin 500mg IV every 6 hours, or 1g every 8 hours  **OR**  -Doripenem 500 mg IV every 8 hours  **Combination therapy:**  -Cefepime 2g IV every 8-12 hours  **OR**  -Ceftazidime 2g IV every 8 hours  **AND** Metronidazole 500 mg IV every 8 hours.  **In case of Beta-lactam allergy:**  -Ciprofloxacin 400 mg IV every 12 hours  **OR**  -Levofloxacin 750 mg IV every 24 hours  **AND** Metronidazole 500 mg IV every 8 hours.  **In case of healthcare acquired, and suspecting:**  **MRSA risk:** Add Vancomycin 15-20 mg/kg IV every 8-12 hours.  **VRE risk:** Add Linezolid or Daptomycin. | | **This includes:**  **•** Patients with sepsis, APACHE II >15, ICU, advanced age, immunocompromised state.  **•** Recommended duration of therapy is 4-7 days, guided by clinical response.  **•** In case of uncomplicated appendicitis/cholecystitis after surgery, then duration is ≤24 hours. |
| **IDSA 2016 Clinical Practice Guidelines for the Management of Adults with Hospital-acquired and Ventilator-associated Pneumonia**  **IDSA 2016 Clinical Practice Guidelines for the Management of Adults with Hospital-acquired and Ventilator-associated Pneumonia**  ***(continued)*** | **Hospital-acquired pneumonia** | | |
|  | -Piperacillin-tazobactam 4.5 g IV every 6 hours  **OR**  -Cefepime or ceftazidime 2 g IV every 8 hours  **OR**  -Levofloxacin 750 mg IV daily  -Ciprofloxacin 400 mg IV every 8 hours  **OR**  -Imipenem 500 mg IV every 6 hours  -Meropenem 1 g IV every 8 hours | **In case of high-risk mortality, prior antibiotic within 90 days, MRSA risk.**  *Use any 2 anti-pseudomonal agents from different classes.*  -Piperacillin-tazobactam 4.5 g IV every 6 hours  **OR**  -Cefepime or ceftazidime 2 g IV every 8 hours  **OR**  -Levofloxacin 750 mg IV daily  -Ciprofloxacin 400 mg IV every 8 hours  **OR**  -Imipenem 500 mg IV every 6 hours  -Meropenem 1 g IV every 8 hours  **OR**  -Amikacin 15–20 mg/kg IV daily  -Gentamicin 5–7 mg/kg IV daily  -Tobramycin 5–7 mg/kg IV daily  **OR**  -Aztreonam 2 g IV every 8 hours  **PLUS**  -Vancomycin 15 mg/kg IV every 8-12 hours  **OR**  -Linezolid 600 mg IV every 12 hours | **•** Oxacillin, nafcillin, and cefazolin are preferred for the  treatment of proven MSSA, but would ordinarily not be used in an empiric regimen for HAP.  **•** If patient has severe penicillin allergy and aztreonam is going to be used instead of any β-lactam–based antibiotic, include coverage for MSSA.  **•** Recommended duration: 7 days, may be extended according to patient’s clinical response. |
|  | **Ventilator-associated Pneumonia** | | |
|  | **Choose two antibiotics with anti-pseudomonal activity in addition to one gram-positive agent.**  *One of the following:*  -Piperacillin-tazobactam 4.5 g IV every 6 hours  **OR**  -Cefepime or ceftazidime 2 g IV every 8 hours  **OR**  -Imipenem 500 mg IV every 6 hours  -Meropenem 1 g IV every 8 hours  **OR**  -Aztreonam 2 g IV every 8 hours  **AND**  *One of the following:*  -Ciprofloxacin 400 mg IV every 8 hours  -Levofloxacin 750 mg IV daily  **OR**  -Amikacin 15–20 mg/kg IV daily  -Gentamicin 5–7 mg/kg IV daily  -Tobramycin 5–7 mg/kg IV daily  **OR**  -Colistin 5mg/kg IV loading dose > 2.5mg × (1.5 × CrCl + 30) IV every 12 hours as maintenance dose.  -Polymyxin B 2.5-3.0 mg/kg/day IV divided in 2 daily doses.  **PLUS**  -Vancomycin 15 mg/kg IV every 8-12 hours  **OR**  -Linezolid 600 mg IV every 12 hours | | **•** Polymyxins should be reserved for settings where there is a high prevalence of multidrug resistance and local expertise in using this medication.  **•** In the absence of other options, it is acceptable to use aztreonam as an adjunctive agent with another β-lactam–based agent because it has different targets within the bacterial cell wall.  **•** Recommended duration: 7 days, may be extended according to patient’s clinical response.  **•** In case of MDR Gram negative susceptible only to polymyxin: Colistin ± inhaled therapy may be recommended as per a specialist. |
| **IDSA 2017 Clinical Practice Guidelines for Healthcare-Associated Ventriculitis and Meningitis**  **IDSA 2017 Clinical Practice Guidelines for Healthcare-Associated Ventriculitis and Meningitis**  ***(continued)*** | **Combination therapy:**  *One of the following.*  -Cefepime 2g IV every every 8 hours  **OR**  -Ceftazidime 2g IV every 8 hours  **OR**  -Meropenem 2g IV every 8 hours  **AND** Vancomycin 15-20mg/kg IV every 8-12 hours  **In case of severe β-lactam allergy:**  -Aztreonam 2g IV every 6-8 hours  **OR**  -Ciprofloxacin 400 mg IV every 8-12 hours  **AND** Vancomycin 15-20mg/kg IV every 8-12 hours | **Recommended antibiotic therapy according to isolated pathogen.**  **MSSA.**  -Nafcillin, or oxacillin  *Alternative therapy:*  -Vancomycin  **MRSA.**  -Vancomycin  *Alternative therapy:*  -Daptomycin, or Linezolid  **Streptococcus pneumoniae.**  ***If Penicillin MIC 0.06 μg/mL****.*  -Penicillin G  *Alternative therapy:*  -Third generation cephalosporin  ***If Penicillin MIC ≥0.12 μg/mL***  > Cefotaxime or ceftriaxone MIC <1.0 μg/mL  -Third generation cephalosporin  *Alternative therapy:*  -Cefepime  **OR**  -Meropenem  **>Cefotaxime or ceftriaxone MIC ≥1.0 μg/mL**  -Vancomycin **AND** third generation cephalosporin  *Alternative therapy:*  -Moxifloxacin  **Pseudomonas aeruginosa.**  -Cefepime, Ceftazidime  **OR**  -Meropenem  *Alternative therapy:*  -Aztreonam  **OR**  -Ciprofloxacin  **Haemophilus influenzae.**  *>β-lactamase negative.*  -Ampicillin  *Alternative therapy:*  -Third generation cephalosporin, cefepime  **OR**  -Fluoroquinolone  *>β-lactamase positive.*  -Third-generation cephalosporin  *Alternative therapy:*  -Cefepime, aztreonam  **OR**  -Fluoroquinolone  **ESBL producing gram negative bacilli.**  -Meropenem  *Alternative therapy:*  -Cefepime  **OR**  -Fluoroquinolone  **Acinetobacter baumannii.**  -Meropenem  *Alternative therapy:*  -Colistin  **OR**  -Polymyxin B  **Other Enterobacteriaceae.**  -Third generation cephalosporin  *Alternative therapy:*  -Meropenem, aztreonam, trimethoprim-sulfamethoxazole, **OR**  -Ciprofloxacin | **•** Recommended duration 10 days. In case of S. aureus, Gram-negative bacilli, continue for 10-14 days.  **•** If CSF cultures persist as positive, continue 10-14 days after last positive culture. |
| **IDSA Clinical Practice Guidelines for the Diagnosis and Management of Skin and Soft Tissue Infections 2014 Update**  **IDSA Clinical Practice Guidelines for the Diagnosis and Management of Skin and Soft Tissue Infections 2014 Update**  ***(continued)***  **IDSA Clinical Practice Guidelines for the Diagnosis and Management of Skin and Soft Tissue Infections 2014 Update**  ***(continued)*** | **Nonpurulent Cellulitis (Mild with no systemic signs)** | | |
|  | -Cephalexin 500 mg PO every 6 hours  **OR**  -Dicloxacillin 500 mg PO every 6 hours  **OR**  -Amoxicillin 500 mg PO every 8 hours  **OR**  -Clindamycin 300-450 mg PO every 6-8 hours |  |  |
|  | **Nonpurulent Cellulitis (Moderate with systemic signs)** | | |
|  | -Penicillin  **OR**  -Cefazolin 1-2g IV every 8 hours  **OR**  -Ceftriaxone 1-2g IV every 8 hours  **OR**  -Clindamycin 600-900 mg IV every 8 hours |  |  |
|  | **Nonpurulent Cellulitis (Severe)** | | |
|  | -Vancomycin 15-20 mg/kg every 8-12 hours  **AND** Piperacillin/Tazobactam 4.5g every 6 hours | -Vancomycin  **AND** Imipenem-cilastatin  **OR**  -Vancomycin  **AND** Meropenem 1g every 8 hours | **•** SIRS, immunocompromised, penetrating trauma, MRSA risk. |
|  | **Necrotizing fasciitis** | | |
|  | -Vancomycin  **AND** Piperacillin-Tazobactam  **AND** Clindamycin | **In case of identified organism.**  **Streptococcus pyogenes.**  -Penicillin G 2-4 MU IV every 4 hours  **AND** Clindamycin 600-900 mg IV every 8 hours  **Aeromonas hydrophila.**  -Doxycycline  **AND** Ciprofloxacin 500 mg IV every 12 hours  **Polymicrobial.**  -Vancomycin  **AND** Piperacillin-Tazobactam  *Alternative therapy:*  -Clindamycin  **OR**  **-** Metronidazole with an aminoglycoside or fluoroquinolone. |  |
|  | **Purulent SSTI (Abscess, Furuncle, Carbuncle) (Moderate)** | | |
|  | -Trimethoprim/Sulfamethoxazole 1-2 DS tablets PO every 12 hours  **OR**  -Doxycycline 100 mg PO every 12 hours | **For MRSA coverage.**  -TMP/SMX  **For MSSA coverage.**  -Dicloxacillin 500 mg PO every 6 hours  **OR**  -Cephalexin 500 mg PO every 6 hours | **•** Incision and drainage are the primary therapy, followed by culture and sensitivity. |
|  | **Purulent SSTI (Abscess, Furuncle, Carbuncle) (Severe)** | | |
|  | -Vancomycin  **OR**  -Daptomycin 4mg/kg IV every 24 hours  **OR**  -Linezolid 600 mg IV/PO every 12 hours  **OR**  -Ceftaroline 600 mg IV every 12 hours | **For MRSA coverage.**  -Refer to Empiric.  **For MSSA coverage.**  -Nafcillin 1-2g IV every 4 hours  **OR**  -Cefazolin 1g IV every 8 hours  **OR**  -Clindamycin 600 mg IV every 8 hours |  |
| **IDSA Guidelines for Diagnosis and Treatment of Adults with Community-acquired Pneumonia**  **IDSA Guidelines for Diagnosis and Treatment of Adults with Community-acquired Pneumonia**  ***(continued)*** | **Outpatient CAP** | | |
|  | **No comorbidities.**  -Amoxicillin 1g PO every 8 hours  **OR**  -Doxycycline 100 mg PO every 12 hours  **OR**  -Macrolide  *Azithromycin 500 mg on first day, then 250 mg PO daily every 24 hours,* ***OR*** *Clarithromycin 500 mg PO every 12 hours* | **In case of comorbidities or risk factors.**  *Combination therapy.*  -Co-Amoxiclav 875/125 mg PO every 12 hours  **OR**  -Cephalosporin *Cefpodoxime 200 mg every 12 hours PO, Cefuroxime 500 mg every 12 hours*  **AND** macrolide or doxycycline  **OR**  - Monotherapy Respiratory fluoroquinolone *Levofloxacin 750 mg, Moxifloxacin 400 mg every 24 hours, Gemifloxacin 320 mg every 24 hours PO* | **•** Risk factors include prior respiratory isolation of MRSA or P. aeruginosa or recent hospitalization  AND receipt of parenteral antibiotics (in the last 90 days).  **•** Comorbidities include chronic heart, lung, liver, or renal disease; diabetes mellitus; alcoholism or  malignancy. |
|  | **Non-severe inpatient pneumonia (Not ICU)** | | |
|  | -ß-lactam  **AND**  Macrolide  **OR**  -Monotherapy Respiratory fluoroquinolone | **In case of prior MRSA isolation.**  -Add either Vancomycin  **OR**  -Linezolid  **In case of prior Pseudomonas isolation.**  *>Add P. aeruginosa coverage.*  -Piperacillin/Tazobactam 4.5 g IV every 6 hours  **OR**  -Cefepime 2g IV every 8 hours  **OR**  -Meropenem 1g IV every 8 hours  **OR**  -Imipenem-cilastatin 500 mg IV every 6 hours | **•** Uncomplicated CAP recommended duration 5 days with the patient being afebrile and clinical stable for 48-72 hours.  **•** De-escalate at 48–72 hours based on cultures and response. |
|  | **Severe inpatient pneumonia (ICU)** | | |
|  | -ß-lactam  **AND** macrolide  **OR**  -ß-lactam  **AND**  A respiratory fluoroquinolone | **In case of prior MRSA isolation.**  -Add either Vancomycin  **OR**  -Linezolid.  **In case of prior Pseudomonas isolation.**  *>Add P. aeruginosa coverage.*  -Piperacillin/Tazobactam 4.5 g IV every 6 hours  **OR**  -Cefepime 2g IV every 8 hours  **OR**  -Meropenem 1g IV every 8 hours  **OR**  -Imipenem-cilastatin 500 mg IV every 6 hours | **•** ESBL coverage should only be considered only on the basis of patient or local microbiological data.  **•** Recommended duration of therapy 5 days, often longer (7-10 days) which is individually tailored according to presence of MRSA/Pseudomonas and patient clinical response.  **•** De-escalate at 48–72 hours based on cultures and response. |
| **IDSA Guidelines for the Management of Encephalitis** | -Acyclovir  **AND** Ceftriaxone 2g IV every 12 hours  **AND** Vancomycin 15-20 mg/kg IV every 8-12 hours  **In case of Listeria risk:**  -Add Ampicillin 2g IV every 4 hours | **Pathogen-directed therapy.**  **Bacterial.**  ***>Listeria monocytogenes.***  -Ampicillin  **AND** gentamicin  *Alternative therapy:*  -TMP/SMX to penicillin-allergic patients.  **Mycobacteria.**  ***>Mycobacterium tuberculosis.***  -4-drug antituberculosis therapy. | **•** Considering adding Ampicillin for Listeria coverage if:   - Age >50 years - Immunocompromised - Pregnancy - Alcohol use disorder   **•** Acyclovir should be started in all suspected encephalitis cases. |
| **IDSA Guidelines on the Diagnosis and Treatment of Diabetes-related Foot Infections**  **IDSA Guidelines on the Diagnosis and Treatment of Diabetes-related Foot Infections**  ***(continued)*** | **Mild infection** | | |
|  | -Dicloxacillin, Nafcillin  **OR**  -1^st^ generation cephalosporin (cephalexin 500 mg PO every 6 hours, cefazolin) | **In case of ß-lactam allergy:**  -Clindamycin 300-450 mg PO every 6-8 hours.  *Recent antibiotic exposure:*  -ß-lactam **or** fluoroquinolone.  **High risk for MRSA:**  -Linezolid, TMP/SMX, Clindamycin, Doxycycline. | **•** Recommended duration 1-2 weeks for mild infections. |
|  | **Moderate or severe** | | |
|  | -Co-Amoxiclav, Ampicillin/Sulbactam 1.5-3g IV every 6 hours  **OR**  -2^nd^, 3^rd^ generation cephalosporins (Cefuroxime, Cefotaxime, Ceftriaxone) | **If patient had recent antibiotic administration.**  **-**Piperacillin/Tazobactam  **OR**  -2^nd^, 3^rd^ generation cephalosporin  **OR**  -Carbapenems  **In case of macerated ulcer or warm climate.**  -Piperacillin/Tazobactam  **AND** Ceftazidime  **OR**  -Ciprofloxacin **AND** Carbapenem  **In case of necrosis and limb ischemia.**  -Co-Amoxiclav, Ampicillin/Sulbactam  **OR**  -Carbapenems (Ertapenem, Meropenem, Imipenem)  **OR**  -2^nd^, 3^rd^ generation cephalosporin  **AND** Clindamycin  **OR**  **-**Metronidazole  **In case of suspected MRSA.**  -Vancomycin, Linezolid  **OR**  -Daptomycin  **In case of suspected ESBL.**  -Carbapenems, fluoroquinolones, Amikacin  **OR**  **-**Colistin | **•** Recommended duration 2-3 weeks for moderate to severe infections.  **•** Extended duration (6 weeks) is needed for osteomyelitis.  **•** After amputation treatment duration is 2-5 days if there is no remaining infected tissue or bone. |
| **IDSA Guidelines on The Practice of Travel Medicine** | **Moderate to severe traveler’s diarrhea** | | |
|  | -Fluoroquinolones Ciprofloxacin 500 mg PO every 12 hours  *In severe/dysentery.*  -Azithromycin 1g mg PO every 24 hours | *Noninvasive diarrhea.*  -Rifaximin 200 mg PO every 8 hours | **•** A minimum of 1-3 days in more severe cases, patient to be re-evaluated 24 hours after beginning therapy. |
|  | **Typhoid fever** | | |
|  | -Ceftriaxone 2g IV every 24 hours | -Ciprofloxacin 500 mg PO every 12 hours | **•** Recommended duration 5-14 days. |
| **IDSA Practice Guidelines for the Management of Bacterial Meningitis** | **2-50 years old**  -Vancomycin 15–20 mg/kg IV every 8-12 hours  **AND** 3^rd^ generation cephalosporin  **50 years old**  -Vancomycin  **AND** Ampicillin 2 g IV every 4 hours  **AND** 3^rd^ generation cephalosporin  **Post-surgery/Head trauma/CSF shunt.**  Vancomycin  *With one of the following.*  -Cefepime 2 g IV every 8 hours  -Ceftazidime 2 g IV every 8 hours  -Meropenem 2 g IV every 8 hours | **Streptococcus pneumoniae.**  ***Penicillin MIC <0.1 µg/mL***  -Penicillin G or Ampicillin  ***Penicillin MIC***  ***0.1-1.0 µg/mL***  -3^rd^ generation cephalosporin  ***Penicillin MIC***  ***≥2 µg/mL***  -Vancomycin  **AND** 3^rd^ generation cephalosporin  **Neisseria meningitidis.**  ***> Penicillin MIC <0.1 µg/mL***  -Penicillin G  **OR**  -Ampicillin  ***>Penicillin MIC***  ***0.1-1.0 µg/mL***  -3^rd^ generation cephalosporin  **Pseudomonas aeruginosa.**  -Cefepime **or** Ceftazidime **or** Meropenem  **MSSA.**  -Nafcillin **or** Oxacillin  **MRSA.**  -Vancomycin | Recommended duration for bacterial meningitidis:   - N.meningitidis 7 days - Strep. Pneumoniae 10-14 days - Strep. Agalactiae 14-21 days - Listeria monocytogenes ≥21 days |
| **IDSA Updates on the Treatment of Drug-Susceptible and Drug-Resistant Tuberculosis** | **Combination therapy.**  -Rifampin 10 mg/kg  -Isoniazid 5 mg/kg  -Pyrazinamide 20–25 mg/kg  -Ethambutol 15–20 mg/kg  For two months, then continuation phase for 4 months.  -Rifampin  **AND** Isoniazid | **In case of HIV coinfection.**  -Same regimen, but rifampin or rifabutin  ***In case of known resistance to isoniazid.***  -Rifampin, Pyrazinamide, Ethambutol  **AND** Moxifloxacin or Levofloxacin  With a continuation phase of 7 months.  ***In case of known resistance to rifampin, and Fluoroquinolone resistance.***  -Bedaquiline  -Pretomanid  -Linezolid |  |
| **Infectious Diseases Society of America 2024 Guidance on the Treatment of Antimicrobial-Resistant Gram-Negative Infections**  **Infectious Diseases Society of America 2024 Guidance on the Treatment of Antimicrobial-Resistant Gram-Negative Infections**  ***(continued)*** | **ESBL-Producing Enterobacterales (Outside the urinary tract)** | | |
|  | -Meropenem 1–2 g IV every 8 hours  -Imipenem-cilastatin 500 mg IV every 6 hours |  |  |
|  | **Carbapenem-Resistant Enterobacterales (KPC producing outside urinary tract)** | | |
|  | - Meropenem-vaborbactam 4 g IV every 8 hours  - Ceftazidime- avibactam 2.5 g IV every 8 hours  - Imipenem-cilastatin-relebactam | -Cefiderocol 2 g IV every 8 hours |  |
|  | **Pseudomonas Aeruginosa with Difficult-To-Treat Resistance (Outside the urinary tract)** | | |
|  | -Ceftolozane-tazobactam 3 g IV every 8 hours  -Ceftazidime-avibactam 2.5 g IV every 8 hours  -Imipenem-cilastatin-relebactam | -Cefiderocol 2 g IV every 8 hours |  |
|  | **Carbapenem-Resistant Acinetobacter Baumannii** | | |
|  | Combination therapy.  -sulbactam-durlobactam  **AND** Carbapenem (Imipenem-Cilastatin or Meropenem) | -High-dose ampicillin–sulbactam 9–12 g/day (sulbactam component)  **AND**  At least one of the following.  -Polymyxin B  -Minocycline  -Tigecycline  -Cefiderocol (refractory cases)  In case sulbactam-durlobactam is not available and as last resort. | **•** Nebulized antibiotics are not suggested for the treatment of respiratory infections caused by CRAB. |
|  | **Stenotrophomonas Maltophilia** | | |
|  | ***Any two of the following agents:***  -Cefiderocol 2 g IV every 8 hours  -Minocycline 200 mg loading, then 100 mg every 12 hours  -TMP/SMX 10–15 mg/kg/day (TMP) divided  -Levofloxacin 750 mg daily | ***Combination therapy.***  -Ceftazidime-avibactam  **AND** Aztreonam |  |
| **SIS IDSA Guidelines on Diagnosing and Managing Complicated Intra-Abdominal Infections in Adults and Children 2016** | **Community acquired mild-to-moderate** | | |
|  | ***Single agents:***  -Cefoxitin  -Ertapenem  -Moxifloxacin  -Tigecycline | ***Combination therapy:***  -Metronidazole  *in combination with one of the following agents:*  -Cefazolin  -Cefuroxime  -Ceftriaxone  -Cefotaxime  -Ciprofloxacin  **OR**  -Levofloxacin | **•** This includes perforated or abscessed appendicitis  and other infections of  mild-to-moderate severity. |
|  | **Community acquired High risk of severe** | | |
|  | ***Single agents:***  -Imipenem-cilastatin  -Meropenem  -Doripenem,  -Piperacillin-tazobactam | ***Combination therapy:***  -Metronidazole  *with one of the following agents:*  - Cefepime  -Ceftazidime  -Ciprofloxacin  **OR**  -Levofloxacin | **•** This includes severe physiologic disturbance,  advanced age,  or immunocompromised state. |
|  | **Health Care–Associated Complicated Intra-Abdominal Infection** | | |
|  | -Carbapenem 1 g IV every 8 hours  -Piperacillin/Tazobactam 4.5 g IV every 6 hours  **OR**  -Metronidazole *with one of the following agents:*  -Ceftazidime  **OR**  -Cefepime | **MRSA risk.**  -Add Vancomycin  **OR**  -Linezolid  **OR**  -Daptomycin  **ESBL/P.aeruginosa risk.**  **ADD** Aminoglycoside | **•** Recommended duration for overall abdominal infections:   - 4 days when source control is complete. - **•** 4-7 days when source control is difficult.   May be extended according to patient’s clinical response and condition. |
|  | **Severe community-acquired acute cholecystitis/ Acute cholangitis of any severity/ Healthcare-associated biliary infection of any severity** | | |
|  | -Metronidazole *in combination with one of the following agents:*  -Imipenem-cilastatin  -Meropenem  -Doripenem  -Piperacillin-tazobactam  -Ciprofloxacin, levofloxacin  -Cefepime | **In case of MRSA risk. *(Only in healthcare acquired).***  **ADD** Vancomycin |  |
| **Surviving Sepsis Campaign: International Guidelines for Management of Sepsis and Septic Shock 2021** | -Beta-lactam  (Piperacillin-tazobactam 4.5 g IV every 6 hours, Cefepime 2 g IV every 8 hours, or Meropenem 1–2 g IV every 8 hours)  **AND** Vancomycin 5–20 mg/kg IV every 8-12 hours or Linezolid, when suspecting MRSA or presence of MRSA risk factors. | **In case of identified source.**  ***Pulmonary***  **ADD** macrolide if CAP overlap.  ***SSTI***  **ADD** Clindamycin if necrotizing infection is suspected.  ***CNS***  **ADD** Ampicillin if listeria risk. | **•** Add MRSA coverage if the following applies:   - Prior positive MRSA culture - High prevalence ICU - Severe septic shock   **•** Consider Pseudomonas coverage if the following applies:   - Healthcare exposure - Prior antibiotic administration - ICU admission   **•** Consider escalating to Carbapenem if:   - ESBL risk - Previous broad spectrum antibiotic administration - Refractory shock   **•** Recommended duration is 7-10 days if the source has been identified and controlled. In case of bacteremia 7-14 days. |

Table S3. Appropriateness of antibiotic therapy stratified by specialty

| **Medical specialty** | **Total, n (%)^a^** | **Patients with appropriate empiric initiation, n (%)**^b^ | **Patients with positive culture, n (%)**^b^ | **Patients receiving inappropriate therapy, n (%)**^b^ |
| --- | --- | --- | --- | --- |
| Internal medicine | 173 (34.6) | 162 (93.6) | 88 (50.9) | 47 (27.2) |
| Neurosurgery | 67 (13.4) | 65 (97.0) | 21 (31.3) | 42 (62.7) |
| ICU | 56 (11.2) | 50 (89.3) | 33 (58.9) | 18 (32.1) |
| General surgery | 41 (8.2) | 40 (97.6) | 20 (48.8) | 12 (29.3) |
| Obstetrics and gynecology | 38 (7.6) | 36 (94.7) | 6 (15.8) | 17 (44.7) |
| Medical oncology | 33 (6.6) | 33 (100) | 12 (36.4) | 7 (21.2) |
| Hematology | 22 (4.4) | 22 (100) | 12 (54.5) | 9 (40.9) |
| Cardiology | 18 (3.6) | 16 (88.9) | 8 (44.4) | 5 (27.8) |
| Thoracic surgery | 11 (2.2) | 11 (100) | 4 (36.4) | 8 (72.7) |
| Neurology | 8 (1.6) | 8 (100) | 4 (50.0) | 1 (12.5) |
| Vascular surgery | 8 (1.6) | 7 (87.5) | 6 (75.0) | 3 (37.5) |
| Plastic surgery | 8 (1.6) | 8 (100) | 2 (25.0) | 5 (62.5) |
| Nephrology | 6 (1.2) | 6 (100) | 4 (66.7) | 0 (0) |
| Palliative care | 3 (0.6) | 3 (100) | 2 (66.7) | 1 (33.3) |
| Otorhinolaryngology | 3 (0.6) | 3 (100) | 1 (33.3) | 2 (66.7) |
| Oral and maxillofacial surgery | 3 (0.6) | 3 (100) | 2 (66.7) | 2 (66.7) |
| Urology | 1 (0.2) | 1 (100) | 1 (100) | 0 (0) |
| Breast surgery | 1 (0.2) | 1 (100) | 0 (0) | 1 (100) |
| **Total** | **500** | **475 (95.0)** | **226 (45.2)** | **180 (36.0)** |

Medical specialties included internal medicine, medical oncology, hematology, cardiology, neurology, nephrology, and palliative care. Surgical specialties included neurosurgery, general surgery, obstetrics and gynecology, thoracic surgery, vascular surgery, plastic surgery, otorhinolaryngology, oral and maxillofacial surgery, urology and breast surgery.

ICU, intensive care unit

^a^Percentages were calculated relative to the total cohort (N = 500).

^b^Percentages were calculated within each specialty using its respective total as the denominator.

***Figure S1.*** *Decision pathway for antibiotic therapy*
